# Enantioselective Recognition of Helicenes by a Tailored Chiral Benzo[ghi]perylene Trisimide π‐Scaffold

**DOI:** 10.1002/anie.202117625

**Published:** 2022-02-18

**Authors:** Ben Teichmann, Ana‐Maria Krause, Mei‐Jin Lin, Frank Würthner

**Affiliations:** ^1^ Institut für Organische Chemie and Center for Nanosystems Chemistry Universität Würzburg Am Hubland 97074 Würzburg Germany; ^2^ State Key Laboratory of Photocatalysis on Energy and Environment College of Chemistry Fuzhou University 350116 China

**Keywords:** Binding Studies, Chirality, Dyes/Pigments, Enantioselectivity, Molecular Recognition

## Abstract

Enantioselective molecular recognition of chiral molecules that lack specific interaction sites for hydrogen bonding or Lewis acid–base interactions remains challenging. Here we introduce the concept of tailored chiral π‐surfaces toward the maximization of shape complementarity. As we demonstrate for helicenes it is indeed possible by pure van‐der‐Waals interactions (π–π interactions and CH–π interactions) to accomplish enantioselective binding. This is shown for a novel benzo[ghi]perylene trisimide (BPTI) receptor whose π‐scaffold is contorted into a chiral plane by functionalization with 1,1′‐bi‐2‐naphthol (BINOL). Complexation experiments of enantiopure (*P*)‐BPTI with (*P*)‐ and (*M*)‐[6]helicene afforded binding constants of 10 700 M^−1^ and 550 M^−1^, respectively, thereby demonstrating the pronounced enantiodifferentiation by the homochiral π‐scaffold of the BPTI host. The enantioselective recognition is even observable by the naked eye due to a specific exciplex‐type emission originating from the interacting homochiral π‐scaffolds of electron‐rich [6]helicene and electron‐poor BPTI.

## Graphical abstract

Helicenes are bound by a newly designed chiral π‐scaffold composed of a perylene trisimide, BINOL and terphenyl subunits. The superb enantiodifferentiation is observable by the naked eye due to the exciplex‐type emission of the homochiral complex.

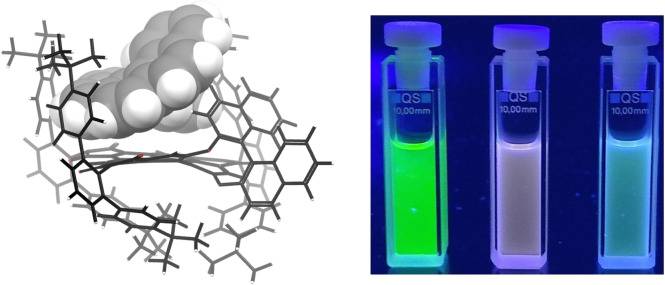

From the early beginnings a main goal in the field of supramolecular chemistry was to accomplish molecular recognition of chiral substrates with high enantioselectivity because this property could entail synthetic receptors with a functionality demonstrated by their natural counterparts, i.e. biological receptors and enzymes. However, such endeavor proved to be very demanding as it requires very well‐tailored supramolecular hosts that make use of several intermolecular interaction sites that provide a chiral space for interaction with the respective substrate. Accordingly, among the plethora of reported supramolecular host‐guest systems the number of examples for enantioselective molecular recognition remains small.^[1]^ And indeed most examples rely on the directionality provided by hydrogen bonds as exemplified by the recognition of amino acid derivatives by crown ethers containing 1,1′‐bi‐2‐naphthol (BINOL) ligands first demonstrated by Cram^[2]^ or the cyclophane‐type carbohydrate receptors introduced more recently by Davis.^[3]^ In this contribution we introduce an alternative strategy that is based on the weak van‐der‐Waals interaction provided by π‐scaffolds. Thus, with the help of the chiral BINOL unit we distort the intrinsically non‐chiral planar π‐scaffold of a benzo[ghi]perylene trisimide (BPTI) into a robust chiral supramolecular host structure. As we will show the chiral environment imparted by this molecule is capable of binding various helicene molecules to afford enantioselective recognition.

The starting point of our study was earlier work in our groups on the distortion of perylene bisimides (PBIs) into chiral scaffolds,^[4]^ molecular recognition of various substrates by PBI cyclophanes^[5]^ and the synthesis of benzo[ghi]perylene trisimides (BPTIs).^[6]^ Remarkably, whilst the nucleophilic substitution of bay‐halogenated PBIs with 1,1′‐bi‐2‐naphthol failed in our earlier research and only the non‐chiral 2,2′‐biphenol could be reacted,^[4]^ this reaction could now be successfully accomplished for the dichlorinated BPTI derivative **6**. As shown in Scheme 1 (for details, see Scheme S1) four literature‐known steps[^6^, ^7^] of esterification, maleic anhydride/*p*‐chloranil bay imidization, saponification and subsequent chlorination afford benzo[ghi]perylene trianhydride **5** in high yield. Also the following step of imidization can be accomplished in good yields for sufficiently reactive aromatic amines such as the commonly applied 2,6‐diisopropylanilin to give BPTIs.^[6]^ However, for our purpose sterically more demanding imide substituents were desirable and accordingly the *tert*‐butyl‐functionalized *meta*‐terphenyl unit was introduced in all three imide positions following our recently introduced base‐assisted imidization protocol with the more reactive amides^[8]^ despite of the lower overall yield of only 18 % accomplished for BPTI **6**. For the subsequent key step of chlorine replacement, standard conditions^[9]^ could be applied to give the racemic BINOL‐functionalized BPTI **7** that could be separated on a chiral stationary phase to give enantiomerically pure (*P*)‐ and (*M*)‐**7**.

**Scheme 1:**
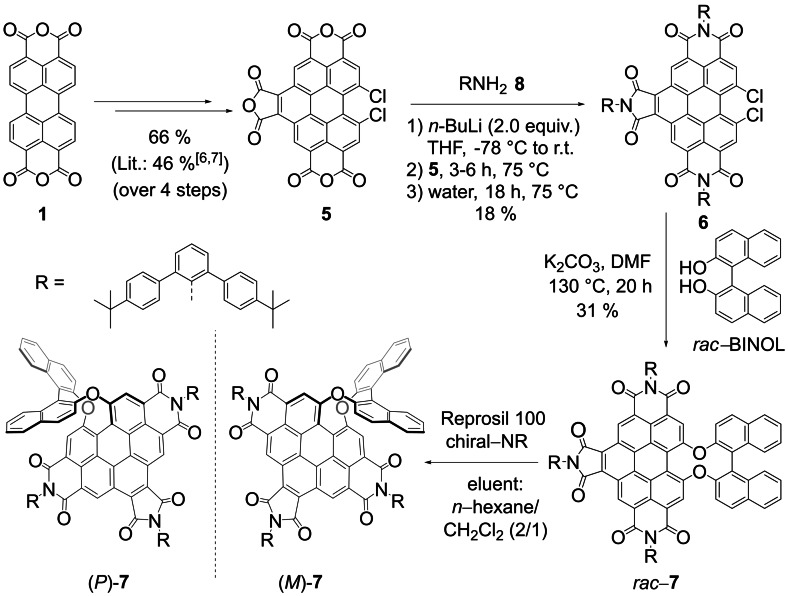
Synthesis of BPTI **7** from perylene dianhydride **1** with separation of the enantiomers on a chiral stationary phase.

To gain structural insights into this new BPTI **7** molecular scaffold, we grew single crystals of the racemic sample suitable for X‐ray analysis (Figure 1).^[10]^ *Rac*‐BPTI **7** crystallizes in the monoclinic crystal system (space group *P*2) with four molecules (two of each enantiomer) per crystal unit (Figure S9c, d). The crystal structure reveals that the arrangement of the large imide substituents is not symmetrical above and below the core of the molecule due to the steric influence of the chiral BINOL group. The outer phenyl rings weakly interact with the main π‐plane of the BPTI. Due to these close contacts on one side (3.6, 3.7 and 4.0 Å) a cavity opens up on the other. It can be clearly seen that the BINOL bridge in the bay position affords a twisting of the π‐surface of the scaffold (Figure 1b zoom) with a dihedral angle of 28.3° (Figure 1a). This dihedral angle matches the one of [6]helicene (27.8°, Figure S10c). Furthermore, the angles between the phenyl planes at the bay position are with 11.2° and 13.4° in good agreement with those of [6]helicene (Figure S10d) and also the coil width of the BPTI scaffold (14.2 Å, Figure S10a) appears to be well suited to accommodate [6]helicene (9.7 Å, Figure S10c). The helical screw sense of the BPTI plane is securely locked by the BINOL group, since the biaryl bridge has to be inverted for an inversion of the helicity.

**Figure 1:**
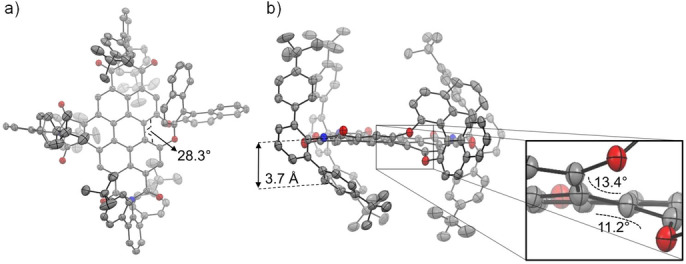
Molecular structure of (*P*)‐BPTI **7** according to single‐crystal X‐ray analysis of a racemic mixture. a) Top view (28.3° as dihedral angle of bay position) and b) side view. In addition, an enlarged excerpt of the bay area is shown to illustrate the chiral helical twist (angles of 13.4° and 11.2° show the rotation of the successive π‐planes to each other). The ellipsoids are set to 50 % probability (C: gray, O: red, N: blue, H: white). Disorder of imides as well as solvent molecules and hydrogen atoms are omitted for clarity. For the packing pattern of (*P*)‐ and (*M*)‐enantiomers, see the Supporting Information (Figure S9).

Thus, by means of appended BINOL unit, enantiomerically pure (*P*)*‐* and (*M*)‐BPTI **7** are expected to show exceptional stability against racemization. Indeed, temperature‐dependent studies on the racemization rate of BPTI **7** in high‐boiling diphenylether (Figure S11) confirm this expectation with an activation barrier of Δ*G* ^≠^_483 K_=160 kJ mol^−1^ (Table S2) that corresponds quite perfectly to the one observed for BINOL (Δ*G* ^≠^_483 K_=165 kJ mol^−^).^[11]^ This value is far above those previously realized for bay‐functionalized PBIs (<120 kJ mol^−1^).[^4^, ^12^] and even [6]helicene (estimated to Δ*G* ^≠^_483 K_=155 kJ mol^−1^)_,_ ^[13]^ thereby demonstrating that BINOL‐functionalized BPTIs are robust chiral scaffolds even at elevated temperatures up to around 150 °C as desired for applications including further chemical derivatization.

Additional characterization of BPTI **7** includes UV/Vis absorption (Figure 2c), circular dichroism (CD, Figure 2a), fluorescence (Figure 2d) and circularly polarized luminescence (CPL, Figure 2b) spectroscopies as well as cyclic voltammetry (Figure S18a) and spectroelectrochemistry (Figure S18b). BPTI **7** exhibits two characteristic fine‐structured absorption bands, S_1_ and S_2_, which cover the spectral range from 280 to 533 nm. The vibronic structure of the S_1_ band resembles those of PBI chromophores with a most intense A_0‐0_ band in methylcyclohexane (MCH) at 502 nm (*ϵ*_max_=30700 M^−1^ cm^−1^). The maximum of the S_2_ absorption band is located at 400 nm. The fluorescence spectra show mirror image behavior to the S_1_ absorption band with *λ*_max_=513 nm and a Stokes shift of 427 cm^−1^ with regard to the absorption maximum of the S_1_ band. Compared to PBIs the fluorescence quantum yield of *Φ*_Fl_=9.4 % in MCH is decreased, presumably due to a quenching by charge transfer from the electron‐rich BINOL substituent. The (*P*)‐ and (*M*)‐enantiomers show a perfect mirror image behavior in the CD (Figure 2a) and CPL spectra (Figure 2b). Due to the rigidity of the distorted BPTI‐BINOL scaffold the CD spectra show a rich pattern of defined narrow bands whose position correlates to bands observed in the absorption spectrum. The *g*_abs_ ((*M*)‐BPTI **7**, 401 nm, Δ*ϵ*=25 M^−1^ cm^−1^, *g*_abs_=0.0007) and *g*_lum_ ((*M*)‐BPTI **7**, 524 nm, *g*_lum_=0.0010) values (Figure S16) are both in the range of 0.001, which is comparable to other chiral molecules of similar size,^[14]^ with a rather low CPL brightness (*B*_CPL_) of 1.62 M^−1^ cm^−1^ (Table S3) similar to e.g. a lot of helicene derivatives.^[15]^ As usual for BPTI dyes, BPTI **7** exhibits three reversible reduction waves at low negative potentials (Figure S18a) which can be assigned to the presence of the three electron‐withdrawing imide groups.^[16]^ The respective UV/Vis spectra of the reduced dye (Figure S18b) are bathochromic shifted from 511 nm (*rac*‐BPTI **7**) to 669 nm (*rac*‐BPTI **7** ^.−^), 691 nm (*rac*‐BPTI **7** ^2−^) and 701 nm (*rac*‐BPTI **7** ^.3−^) in dimethylformamide.

**Figure 2:**
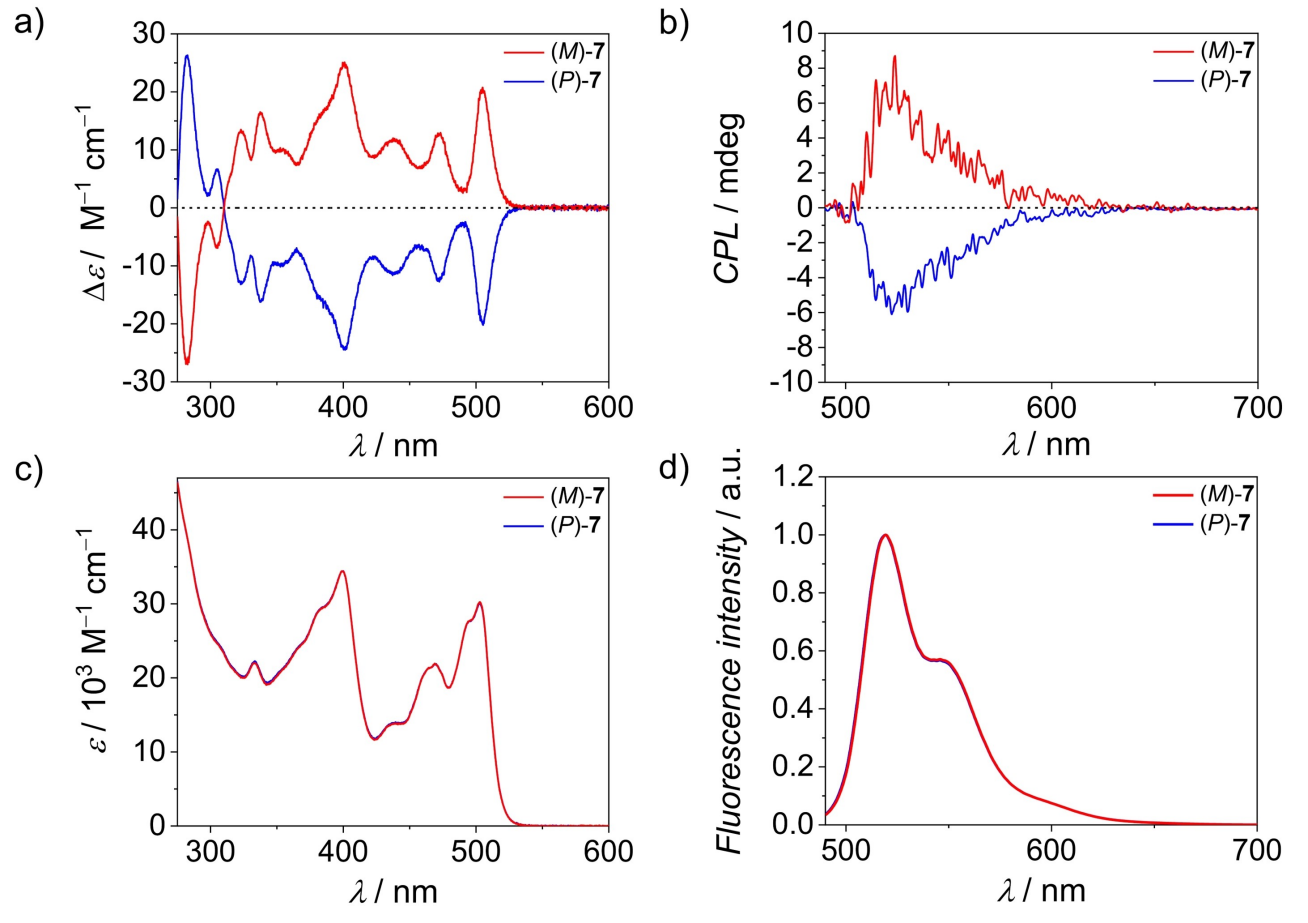
a) CD (*c*≈10^−5^ M), b) CPL (*c*≈10^−7^ M), c) UV/Vis absorption (*c*≈10^−5^ M) and d) fluorescence (c≈10^−7^ M) spectra of (*M*)‐BPTI **7** (red line; *λ*_ex_=400 nm) and (*P*)‐BPTI **7** (blue line; *λ*_ex_=400 nm) in MCH at 293 K.

The molecular structure of BPTI **7** observed in the crystal (Figure 1) shows a rather rigid BINOL‐BPTI framework with one naphthol unit being positioned edge‐on above and the other naphthol unit being stretched away from the BPTI π‐scaffold. The location of the former one most likely is responsible for the significantly quenched BPTI fluorescence because similar conformation‐dependent fluorescence quenching by electron‐rich aryloxy groups has been observed before for various PBIs.^[17]^ With regard to the binding of chiral π‐systems the position of this naphthol unit appears to be quite ideal because it might provide additional CH‐π‐interactions with protons of aromatic guest molecules. Thus, we envisioned that BPTI **7** might indeed be suitable to form supramolecular complexes even with pristine chiral π‐scaffolds such as helicenes,^[18]^ which have received attention in chiral supramolecular chemistry in recent years^[19]^ and lack any functional groups for hydrogen bonding or other specific directional noncovalent interactions. However, with the newly presented host BPTI **7** they might be bound, due to the high shape complementarity pointed out above. DFT calculations^[20]^ for the complex with [6]helicene looked indeed quite promising (Figure 3a). Thus, as revealed by these calculations, there is a reasonably large π‐π‐contact surface between homochiral BPTI and [6]helicene that screens the chiral distortion, and furthermore there are CH‐π‐interactions between the peripheral substituents of BPTI and the protons of the [6]helicene. In particular the *meta*‐terphenyl substituents attached to the three imide units seem to act like molecular hinges that allow the adjustment of the geometry of the binding site to the respective guest molecule whilst closing the space available on the opposing site of the BPTI framework.

**Figure 3:**
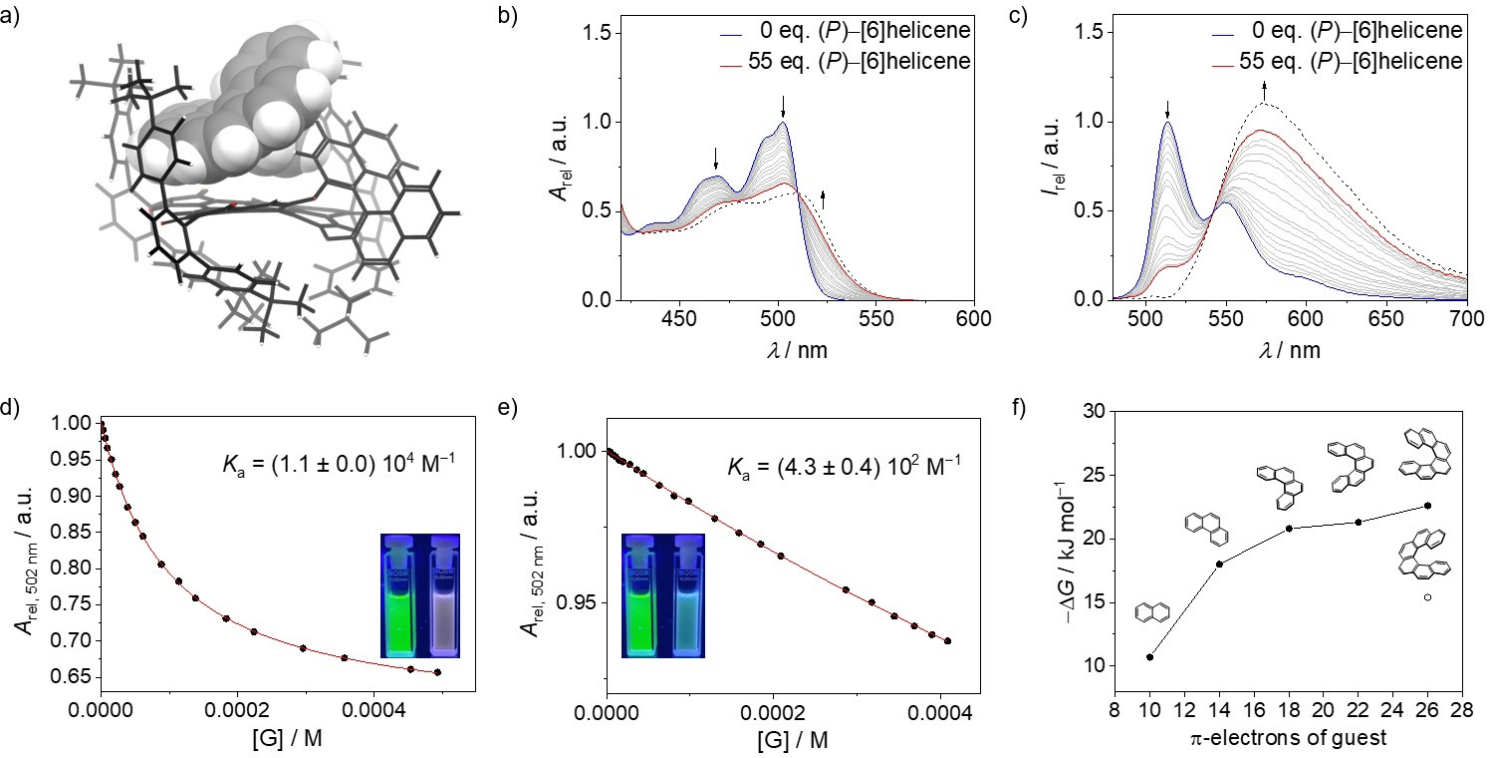
a) DFT‐optimized geometry for the 1 : 1 complex of (*P*)‐BPTI **7** and (*P*)‐[6]helicene at the wb97xd/6‐31 g(d) level of theory. b), c) UV/Vis and fluorescence titration experiments for (*P*)‐BPTI **7** host (blue line) upon addition of 55 equiv (*P*)‐[6]helicene guests (red line) in MCH at 293 K (black dashed line: calculated spectrum for the pure 1 : 1 complex (see the Supporting Information)). d), e) Data points measured at 502 nm for the titration experiment of (*P*)‐BPTI **7** upon addition of (*P*)‐[6]helicene and (*M*)‐[6]helicene, respectively, with the nonlinear curve fit according to the 1 : 1 binding model. The insets show photographs taken under UV light of the free (*P*)‐BPTI **7** solution (left) and (*P*)‐BPTI **7** after addition of the respective (*P*)‐ and (*M*)‐[6]helicene (right). f) Plot for the experimental Gibbs free energies for the BPTI‐helicene complexes versus the number of π‐electrons of the helicene guest molecules.

Motivated by these calculations we carried out UV/Vis and fluorescence titration experiments at a constant concentration of (*P*)‐BPTI host with (*P*)‐[6]helicene guest in MCH at room temperature. As shown in Figure 3b, c upon addition of an excess of guest molecules a nice coverage of the transition from unbound (*P*)‐BPTI **7** to the 1 : 1 complex with (*P*)‐[6]helicene (calculated black dashed lines) could be accomplished and a substantial binding constant of 10 700 M^−1^ could be derived by global data analysis (for details, see the Supporting Information).^[21]^ In contrast, the related titration experiments for (*P*)‐BPTI with (*M*)‐[6]helicene (Figures S25, S27) afforded only minor spectral changes due to an about twenty‐fold smaller binding constant (*K*=550 M^−1^, Table S4). These values relate to Gibbs energies of 22.6 and 15.4 kJ mol^−1^ (Table S4) for the respective diastereomeric complexes, thereby confirming the pronounced enantiomer discrimination imparted by the chiral host. Due to the fact that the complexes between homochiral π‐surfaces of BPTI **7** and [6]helicene show pronounced charge transfer character with exciplex‐type strongly red‐shifted emission (Figure 3c), the enantiomer discrimination can be observed by the naked eye (Figure 3d, e insets).

For a better understanding of the [6]helicene interaction with BPTI **7**, a NMR titration in a solvent mixture of MCH‐*d*_14_ and toluene‐*d*_8_ (24 : 1) was performed (Figure S30). Here, characteristic protons of the BPTI scaffold as well as of the BINOL unit exhibit highfield shifts of up to 0.38 ppm. By fitting these shifts with the 1 : 1 model a binding constant of 3000 M^−1^ could be determined that is in good accordance to the other titrations if we consider the competitive character of the aromatic solvent toluene‐*d*_8_ that we employed for solubility reasons. It is worth mentioning that the proton of the naphthol unit that is inclined above the BPTI scaffold undergoes exactly the same shift upon [6]helicene complexation as the one of the closely located BPTI scaffold (0.38 ppm), thereby highlighting the embedment of [6]helicene in this cleft‐like binding pocket. This result confirms our structural model derived from DFT calculations (Figure 3a) that shows how [6]helicene is hooked into the scaffold of the receptor like a screw into its nut. Thereby, an impressive enantioselective recognition of a chiral π‐scaffold by pure van‐der‐Waals interactions is achieved by the high shape complementarity between the π‐planes of BPTI and [6]helicene and additional supporting CH–π interactions between helicene protons and the π‐planes of the *meta*‐terphenyl and the BINOL subunits (Figure 3a).

In our subsequent studies we also elucidated the binding strength of BPTI **7** for the lower helicene congeners [5]helicene, [4]helicene, phenanthrene as well as naphthalene. Notably, among those only [5]helicene can be separated into its (*P*)‐ and (*M*)‐enantiomers. However, due to a low activation barrier for racemization of around 100 kJ mol^−1[5c, 22]^ for this molecule the determination of binding constants for the respective (*P*)‐ and (*M*)‐enantiomers proved to be elusive. Accordingly, for the whole series of guest molecules we determined the binding constants for the racemic BPTI with the various [*n*]helicenes under the assumption that always complexes consisting of homochiral BPTI and helicene guest molecules are formed (for details, see Figures S19–S23). Evaluation of the binding isotherms in MCH at room temperature afforded binding constants ranging from 80 M^−1^ for naphthalene, 1600 M^−1^ for phenanthrene, 5200 M^−1^ for [4]helicene up to 6300 M^−1^ for [5]helicene (Table S4). The steep increase in the corresponding Gibbs free binding energies between naphthalene and phenanthrene (Figure 3f) suggests that up to three benzene rings of the aromatic guests may tightly interact with the BPTI π‐plane whilst some additional CH‐π‐interactions between the larger helicenes and the peripheral terphenyl and naphthol subunits provide some additional binding strength.

In conclusion, we have introduced a uniquely tailored molecular receptor for helicene guest molecules that enabled a pronounced enantiodiscrimination that could be sensed by the naked eye. This enantiodiscrimination originated from the shape complementarity of a benzo[ghi]perylene trisimide (BPTI) whose π‐scaffold was contorted into a chiral configuration by a BINOL substituent that enforced a helical twist of the neighboring BPTI backbone. Helicene binding by this unique and up to high temperature stable chiral backbone is further supported by three *meta*‐terphenyl imide substituents that enclose the helicene guest molecules by additional CH–π interactions. Our next goal is to explore these chiral BPTI scaffolds for supramolecular chirality sensing of natural products^[23]^ and for enantioselective supramolecular catalysis.^[24]^

## Conflict of interest

The authors declare no conflict of interest.

## Supporting information

As a service to our authors and readers, this journal provides supporting information supplied by the authors. Such materials are peer reviewed and may be re‐organized for online delivery, but are not copy‐edited or typeset. Technical support issues arising from supporting information (other than missing files) should be addressed to the authors.

Supporting Information

Supporting Information

Supporting Information

## Data Availability

The data that support the findings of this study are available from the corresponding author upon reasonable request.
