# Supplementary material for: Enantioselective Recognition of Helicenes by a Tailored Chiral Benzo[ghi]perylene Trisimide π‐Scaffold
Source: Angew Chem Int Ed Engl. 2022 Feb 18;61(15):e202117625. doi: 10.1002/anie.202117625 (PMC9303377; doi:10.1002/anie.202117625)

checkCIF/PLATON report

Structure factors have been supplied for datablock(s) bt50_ab_sq

THIS REPORT IS FOR GUIDANCE ONLY. IF USED AS PART OF A REVIEW PROCEDURE FOR PUBLICATION, IT SHOULD NOT REPLACE THE EXPERTISE OF AN EXPERIENCED CRYSTALLOGRAPHIC REFEREE.

No syntax errors found.   CIF dictionary   Interpreting this report

Datablock: bt50_ab_sq

Bond precision: C-C = 0.0031 A   Wavelength=1.54178

Cell:	a=13.8382(5)	b=30.2689(11)	c=26.5850(9)
	alpha=90	beta=92.719(2)	gamma=90
Temperature:	100 K		

	Calculated	Reported
Volume	11123.1(7)	11123.0(7)
Space group	P 21/n	P 21/n
Hall group	-P 2yn	-P 2yn
Moiety formula	C126 H103 N3 O8, 2(C2 H3 N) [+ solvent]	C126 H103 N3 O8, 2(C2 H3 N) [+ SOLVENT]
Sum formula	C130 H109 N5 O8 [+ solvent]	C130 H109 N5 O8
Mr	1869.23	1869.22
Dx,g cm-3	1.116	1.116
Z	4	4
Mu (mm-1)	0.541	0.541
F000	3952.0	3952.0
F000’	3963.05	
h,k,lmax	17,37,32	17,37,32
Nref	22105	22047
Tmin,Tmax	0.897,0.937	0.702,0.754
Tmin’	0.873	

Correction method= # Reported T Limits: Tmin=0.702 Tmax=0.754
AbsCorr = MULTI-SCAN

Data completeness= 0.997   Theta(max)= 72.603

R(reflections)= 0.0586(18384)   wR2(reflections)= 0.1598(22047)

S = 1.038   Npar= 1365

The following ALERTS were generated. Each ALERT has the format
test-name_ALERT_alert-type_alert-level.
Click on the hyperlinks for more details of the test.

Alert level C

```
PLAT220_ALERT_2_C NonSolvent   Resd 1  C   Ueq(max)/Ueq(min) Range       3.8 Ratio
PLAT222_ALERT_3_C NonSolvent Resd 1  H   Uiso(max)/Uiso(min) Range       4.6 Ratio
PLAT230_ALERT_2_C Hirshfeld Test Diff for    C73      --C74      .       7.0 s.u.
PLAT230_ALERT_2_C Hirshfeld Test Diff for    C122     --C123     .       6.0 s.u.
PLAT242_ALERT_2_C Low    'MainMol' Ueq as Compared to Neighbors of       C73 Check
PLAT242_ALERT_2_C Low    'MainMol' Ueq as Compared to Neighbors of      C103 Check
PLAT244_ALERT_4_C Low    'Solvent' Ueq as Compared to Neighbors of      C1_2 Check
PLAT906_ALERT_3_C Large K Value in the Analysis of Variance ......     4.140 Check
PLAT911_ALERT_3_C Missing FCF Refl Between Thmin & STh/L=    0.600         6 Report
```

Alert level G

```
FORMU01_ALERT_1_G  There is a discrepancy between the atom counts in the
            _chemical_formula_sum and _chemical_formula_moiety. This is
            usually due to the moiety formula being in the wrong format.
            Atom count from _chemical_formula_sum:   C130 H109 N5 O8
            Atom count from _chemical_formula_moiety:C126 H103 N3 O8
PLAT002_ALERT_2_G Number of Distance or Angle Restraints on AtSite        24 Note
PLAT003_ALERT_2_G Number of Uiso or Uij Restrained non-H Atoms ...        22 Report
PLAT083_ALERT_2_G SHELXL Second Parameter in WGHT  Unusually Large      5.74 Why ?
PLAT175_ALERT_4_G The CIF-Embedded .res File Contains SAME Records         3 Report
PLAT176_ALERT_4_G The CIF-Embedded .res File Contains SADI Records         4 Report
PLAT177_ALERT_4_G The CIF-Embedded .res File Contains DELU Records         3 Report
PLAT178_ALERT_4_G The CIF-Embedded .res File Contains SIMU Records         3 Report
PLAT301_ALERT_3_G Main Residue  Disorder ..............(Resd  1  )        6% Note
PLAT412_ALERT_2_G Short Intra XH3 .. XHn     H35      ..H40D     .      2.09 Ang.
                                                      x,y,z  =     1_555 Check
PLAT412_ALERT_2_G Short Intra XH3 .. XHn     H89      ..H96A     .      2.13 Ang.
                                                      x,y,z  =     1_555 Check
PLAT413_ALERT_2_G Short Inter XH3 .. XHn     H124     ..H95D     .      2.11 Ang.
                                          1/2-x,1/2+y,1/2-z  =     2_555 Check
PLAT606_ALERT_4_G Solvent Accessible VOID(S) in Structure ........         ! Info
PLAT720_ALERT_4_G Number of Unusual/Non-Standard Labels ..........        12 Note
PLAT860_ALERT_3_G Number of Least-Squares Restraints .............       323 Note
PLAT869_ALERT_4_G ALERTS Related to the Use of SQUEEZE Suppressed          ! Info
PLAT912_ALERT_4_G Missing # of FCF Reflections Above STh/L=  0.600        52 Note
PLAT978_ALERT_2_G Number C-C Bonds with Positive Residual Density.         1 Info
```

0 **ALERT level A** = Most likely a serious problem - resolve or explain
0 **ALERT level B** = A potentially serious problem, consider carefully
9 **ALERT level C** = Check. Ensure it is not caused by an omission or oversight
18 **ALERT level G** = General information/check it is not something unexpected

1 ALERT type 1 CIF construction/syntax error, inconsistent or missing data
12 ALERT type 2 Indicator that the structure model may be wrong or deficient
5 ALERT type 3 Indicator that the structure quality may be low
9 ALERT type 4 Improvement, methodology, query or suggestion
0 ALERT type 5 Informative message, check

It is advisable to attempt to resolve as many as possible of the alerts in all categories. Often the minor alerts point to easily fixed oversights, errors and omissions in your CIF or refinement strategy, so attention to these fine details can be worthwhile. In order to resolve some of the more serious problems it may be necessary to carry out additional measurements or structure refinements. However, the purpose of your study may justify the reported deviations and the more serious of these should normally be commented upon in the discussion or experimental section of a paper or in the "special_details" fields of the CIF. checkCIF was carefully designed to identify outliers and unusual parameters, but every test has its limitations and alerts that are not important in a particular case may appear. Conversely, the absence of alerts does not guarantee there are no aspects of the results needing attention. It is up to the individual to critically assess their own results and, if necessary, seek expert advice.

Publication of your CIF in IUCr journals

A basic structural check has been run on your CIF. These basic checks will be run on all CIFs submitted for publication in IUCr journals (*Acta Crystallographica*, *Journal of Applied Crystallography*, *Journal of Synchrotron Radiation*); however, if you intend to submit to *Acta Crystallographica Section C* or *E* or *IUCrData*, you should make sure that full publication checks are run on the final version of your CIF prior to submission.

Publication of your CIF in other journals

Please refer to the *Notes for Authors* of the relevant journal for any special instructions relating to CIF submission.

PLATON version of 05/12/2020; check.def file version of 05/12/2020

Datablock bt50_ab_sq - ellipsoid plot

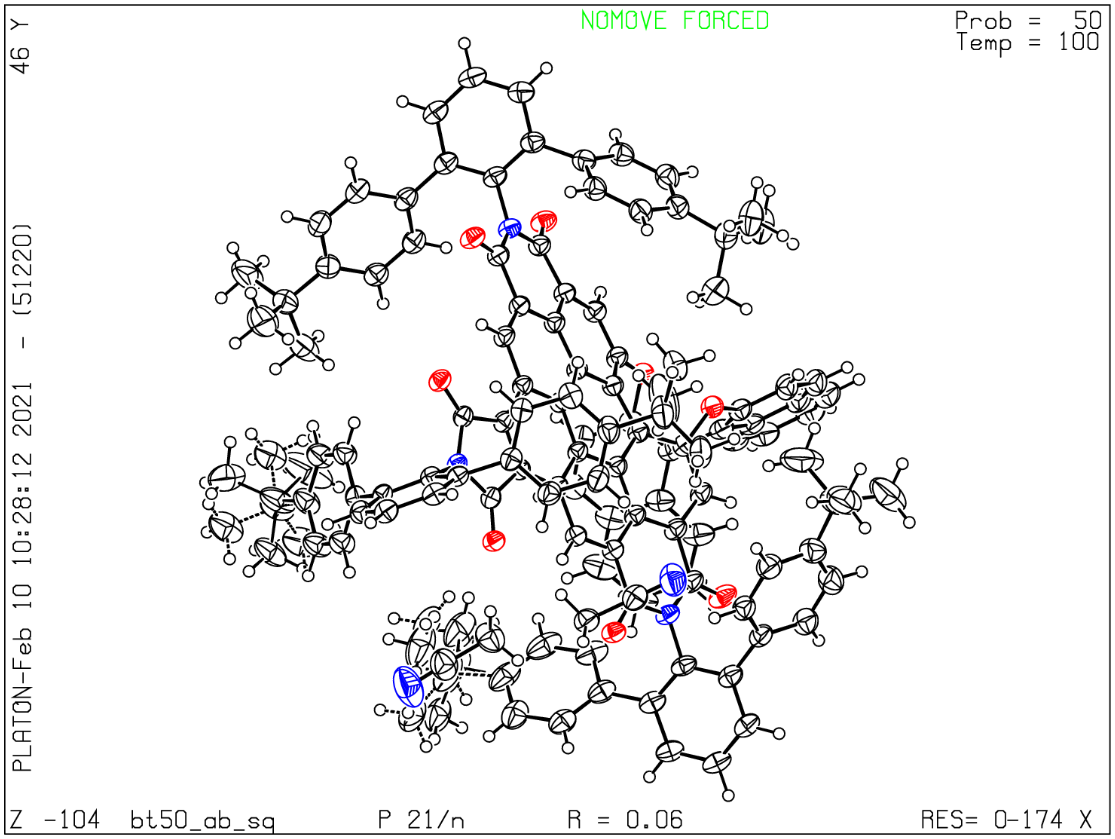

Supplement: Supplementary file 2 — Supporting Information [file ANIE-61-0-s003.pdf]
