# Supplementary material for: Enantioselective Recognition of Helicenes by a Tailored Chiral Benzo[ghi]perylene Trisimide π‐Scaffold
Source: Angew Chem Int Ed Engl. 2022 Feb 18;61(15):e202117625. doi: 10.1002/anie.202117625 (PMC9303377; doi:10.1002/anie.202117625)
Supplement: Supplementary file 3 — Supporting Information [file ANIE-61-0-s002.pdf]

Angewandte Chemie
Eine Zeitschrift der Gesellschaft Deutscher Chemiker

Supporting Information

Enantioselective Recognition of Helicenes by a Tailored Chiral Benzo[ghi]perylene Trisimide π-Scaffold

B. Teichmann, A.-M. Krause, M.-J. Lin, F. Würthner**

Table of Contents

Experimental Procedures S3
Synthesis and Compound Characterization S4
NMR Spectra S7
Mass Spectra S9
HPLC Separation of the Enantiomers S10
Single Crystal X-ray Analysis S11
Activation Parameters for the Racemization of BPTI 7 .... S13
Optical Spectroscopy S16
(Spectro-) Electrochemical Properties S19
Titration Studies S20
Supporting References S28

Experimental Procedures

Chemicals: All chemicals and solvents were purchased from commercial suppliers and used without further purification. Anhydrous solvents were dispensed from a solvent purification system (Innovative Technology). Perylene-3,4,9,10-tetracarboxylic tetraethyl ester (**2**), benzo[ghi]perylene 1,2-anhydride-4,5,10,11-tetracarboxylic tetraethyl ester (**3**), benzo[ghi]perylene 1,2,4,5,10,11-hexacarboxylic trisanhydride (**4**), benzo[ghi]perylene-1,12-dichloro-3,4,6,7,9,10-hexacarboxylic trisanhydride (**5**) and 2,6-bis(4-*tert*-butylphenyl)aniline (**8**) were synthesized according to literature known procedures.[S1-S3] *Rac*-[5]helicene (**G4**) was synthesized in a similar procedure as reported in literature[S4] with (*E*/*Z*)-1,2-bis(2-napthyl)ethylene (200 mg, 713 μmol), iodine (200 mg, 1.58 mmol) and propylene oxide (8.59 g, 148 mmol), dissolved in cyclohexane and irradiated with UV/vis light (λ = 254–575 nm) for 3 h. *Rac*-[6]helicene (**G5**) was synthesized in a similar procedure as reported in literature[S5] with (*E*/*Z*)-2,7-bis(2-phenylvinyl)naphthalene (100 mg, 301 μmol) and iodine (200 mg, 1.58 mmol), dissolved in toluene and irradiated with UV/vis light (λ = 254–575 nm) for 20 h. The resolution of the enantiomers of [6]helicene could be achieved by chiral HPLC (eluent: *n*-hexane/CH_2Cl_2 = 4/1 v/v %, flow rate 6.5 mL/min). The enantiomeric excess was determined by analytical HPLC (Reprosil 100 Chiral-NR 8 μm, Trentec, eluent: *n*-hexane/CH_2Cl_2 = 4/1 v/v %, flow rate 1 mL/min) with values of ee > 99 %. Before and after each experiment with enantiomers the ee value of the respective enantiomer was checked via analytical HPLC.

High performance liquid chromatography (HPLC): Analytical HPLC was carried out on a JASCO system (PU 2080 PLUS) with a diode array detector (MD 2015), equipped with a ternary gradient unit (DG-2080-533) and inline-degasser (LG 2080-02). Recycling semipreparative HPLC was carried out on a JAI LC- 9105. Chiral resolution was carried out using a Trentec Reprosil-100 Chiral-NR 8 μm-column.

Gel permeation chromatography (GPC): GPC was performed on a Shimadzu Recycling GPC-System (LC-20AD Prominence Pump; SPDMA20A Prominence Diode Array Detector) with three or two preparative columns (Japan Analytical Industries Co., Ltd.; JAIGEL-1 H, JAIGEL-2H and JAIGEL-2.5 H) in chloroform (HPLC grade, stabilized with 0.1 % ethanol) with a flow rate of 6.5 or 5.0 mL/min.

NMR spectroscopy: ^{1}H NMR and ^{13}C NMR spectra were recorded on a Bruker Avance III HD 400 spectrometer at 295 K. Chemical shift data are reported in parts per million (ppm, δ scale) and referenced internally to the residual proton (for proton NMR) in the solvent (CD_2Cl_2: δ = 5.32) or to the carbon resonance (CD_2Cl_2: δ = 53.84). The coupling constants are listed in Hertz.

Mass spectrometry: High-resolution ESI TOF spectra were acquired on a Bruker Daltonics microTOF focus spectrometer.

Melting points: Melting points were measured with the melting point apparatus SMP50 (Stuart / Coal-Parmer) and are uncorrected.

Optical UV/vis absorption spectroscopy: All spectroscopic measurements were carried out at 293 K using solvents of spectroscopic grade. The absorption spectra were recorded on a JASCO V-770 or V-670 spectrometer equipped with a PAC-743R Peltier for temperature control. The samples were measured in conventional UV/vis and fluorescence quartz cell (Hellma Analytics) with 10 mm path length.

Steady-state fluorescence spectroscopy: Fluorescence spectra were recorded on an Edinburgh Instruments FLS980-D2D2-ST spectrometer and were corrected against the photomultiplier sensitivity and the lamb intensity. The quantum yield was determined as average value of four different excitation wavelengths (λ_{ex} = 440 nm; 445 nm; 450 nm; 455 nm) relative to *N*,*N*'-bis(2,6-diisopropylphenyl)-3,4:9,10-bis(dicarboxdiimide) (Φ_{Fl} = 100 % in $CHCl_3$)[S6] as a standard under highly diluted (OD ≤ 0.05) and magic angle (54.7°) conditions. Fluorescence lifetimes were determined with an EPL picosecond pulsed diode laser (λ_{ex} = 403.8 nm) for time-correlated single-photon counting (TCSPC) with an Edinburgh Instruments FLS980-D2D2-ST spectrometer. The samples were measured in conventional fluorescence quartz cells (Hellma Analytics) with 10 mm path length.

Ciruclar dichroism (CD) spectroscopy: CD spectra were measured with a Jasco J-810 spectropolarimeter equipped with a JASCO CDF-426S Peltier temperature controller and with a customized JASCO CPL-300/J-1500 hybrid spectrometer. The samples were measured in conventional UV/vis and fluorescence quartz cells (Hellma Analytics) with 10 mm path length.

Circular polarized luminescence (CPL) spectroscopy: CPL spectra were recorded with a customized JASCO CPL-300/J-1500 hybrid spectrometer. The HAT photomultiplier current was 750 V and the spectra were recorded with a scan speed of 100 nm/min and a D.I.T. of 2 sec. 30 accumulations were measured. The excitation and emission bandwidth were set to 30 nm and 15 nm. The samples were measured in conventional fluorescence quartz cells (Hellma Analytics) with 10 mm path length.

Single crystal X-ray analysis: The diffraction images for X-ray crystallographic analysis were collected on a Bruker D8 Quest Kappa diffractometer with a Photon II CPAD area detector using Cu Kα radiation. The structure was solved using direct methods, expanded with Fourier techniques, and refined with the SHELX software package.[S7] All non-hydrogen atoms were refined anisotropically. Hydrogen atoms were included in the structure factor calculation on geometrically idealized positions. Crystallographic data have been deposited with the Cambridge Crystallographic Data Centre as supporting publication CCDC2132523. These data can be obtained free of charge from The Cambridge Crystallographic Data Centre via www.ccdc.ac.uk/data.request/cif.

Synthesis and Compound Characterization

The syntheses of precursors **2**, **3**, **4**, **5** and **8** are described in the literature.[S1-S3]

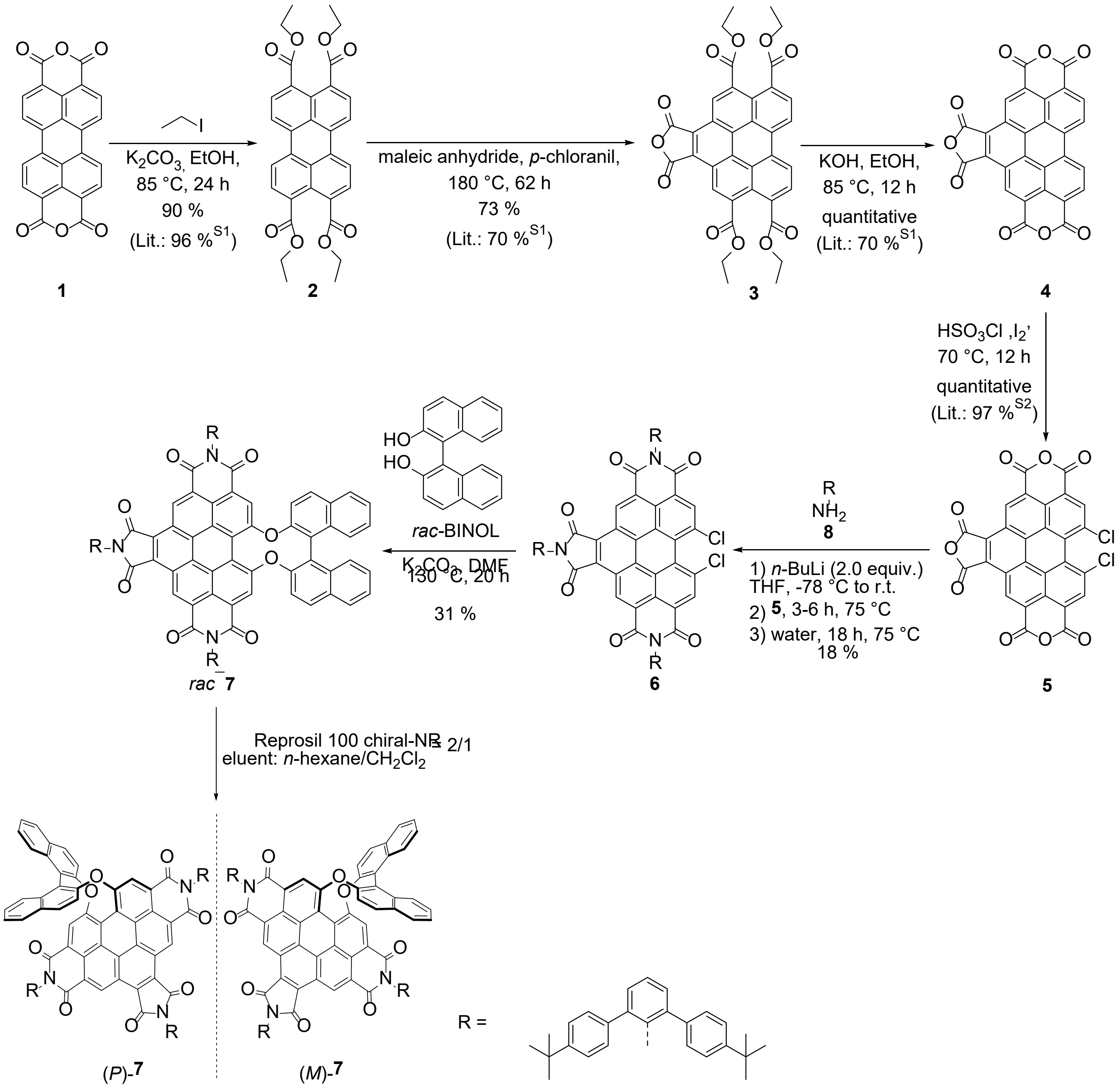

Scheme S1. Synthesis of literature known precursor **5** and new BPTI **6** and BPTI **7**, with EtOH as ethanol, *n*-BuLi as *n*-butyllithium, THF as tetrahydrofuran and DMF as *N*, *N*-dimethylformamide.

Synthesis of benzo[ghi]perylene-1,12-dichloro-3,4,6,7,9,10-hexacarboxylic tris(2,6-di(4-*tert*-butylphenyl)phenyl)imide (6)

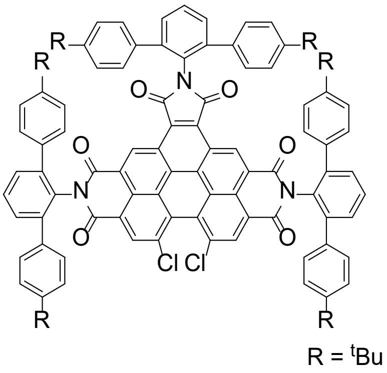

Under a nitrogen atmosphere, 2,6-bis(4-*tert*-butylphenyl)-aniline (**8**) (436 mg, 1.22 mmol, 3.0 mol. eq.) was dissolved in dry THF (14 mL) in a pressure Schlenk tube and cooled down to −78 °C. Subsequently, *n*-BuLi in *n*-hexane (1.50 mL, 1.60 M, 2.40 mmol, 5.9 mol eq.) was added and the reaction mixture stirred at −78°C for 1 h. The mixture was allowed to warm up to room temperature and benzo[ghi]perylene-1,12-dichloro-3,4,6,7,9,10-hexacarboxylic trisanhydride (**5**) (227 mg, 409 μmol, 1.0 mol eq.) was added. The solution was stirred for 4 h at 75 °C and subsequently water (70 mL) was added and the solution stirred for another 18 h at 75 °C. The reaction mixture was allowed to cool to room temperature, THF was evaporated under reduced pressure and 1 M hydrochloric acid solution (50 mL) was added. The reaction mixture was extracted with CH_2Cl_2 (5 x 30 mL), the combined organic phases were washed multiple times with sodium carbonate solution and water, dried over Na_2SO_4, filtered and the solvent was removed in *vacuo*. The crude product was purified by column chromatography (eluent: *n*-hexane/CH_2Cl_2 = 1/2 v/v %) and recycling GPC ($CHCl_3$) to yield compound **6** as a brown-yellow solid (114 mg, 72.4 μmol, 18 %). **Mp.**: >300 °C. **^{1}H NMR** (400 MHz, CD_2Cl_2): *δ* (ppm) = 10.02 (s, 2H, Ar-*H*), 8.88 (s, 2H, Ar-*H*), 7.70 (m, 3H, Ar-*H*), 7.55 (m, 6H, Ar-*H*), 7.33 (m, 8H, Ar-*H*), 7.21 (m, 12H, Ar-*H*), 7.09 (m, 4H, Ar-*H*), 1.06 (s, 18H, Alkyl-C*H*$_3$), 1.00 (s, 18H, Alkyl-C*H*$_3$), 0.93 (s, 18H, Alkyl-C*H*$_3$). **^{13}C NMR** (101 MHz, CD_2Cl_2): *δ* (ppm) = 168.3 (C_q), 163.6 (C_q), 163.3 (C_q), 150.9 (C_q), 150.79 (C_q), 150.78 (C_q), 143.5 (C_q), 142.2 (C_q), 141.9 (C_q), 136.3 (C_q), 136.2 (*C*H), 136.1 (C_q), 135.9 (C_q), 132.8 (*C*H), 131.5 (C_q), 130.9 (*C*H), 130.8 (*C*H), 130.59 (*C*H), 130.55 (*C*H), 129.83, 129.80 (*C*H), 129.6 (*C*H), 128.3 (*C*H), 128.1 (C_q), 127.6 (C_q), 127.2 (C_q), 126.7 (C_q), 125.8 (*C*H), 125.6 (*C*H), 125.3 (*C*H), 125.0 (C_q), 124.8 (C_q), 123.9 (C_q), 123.4 (C_q), 53.8, 34.62 (C_q), 34.59 (C_q), 34.5 (C_q), 31.22 (*C*H$_3$), 31.15 (*C*H$_3$), 31.1 (*C*H$_3$). **HRMS** (ESI, positive mode, MeCN/$CHCl_3$): *m/z* calcd for $C_{106}H_{91}Cl_2N_3NaO_6^+$ [M + Na]$^+$: 1594.61771 , found: 1594.59825.

Synthesis of BINOL-functionalized BPTI 7

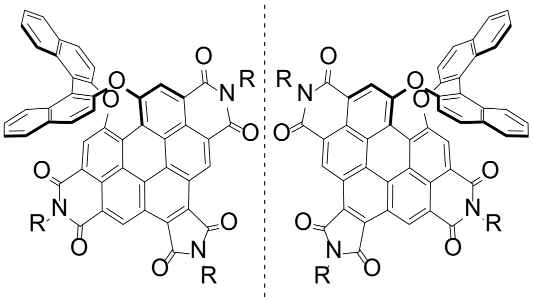

(*P*)-**7** (*M*)-**7**

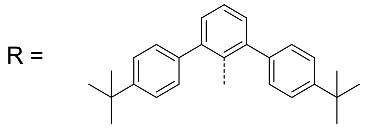

Under a nitrogen atmosphere, benzo[ghi]perylene-1,12-dichloro-3,4,6,7,9,10-hexa-carboxylic tris(2,6-di(4-*tert*-butylphenyl)phenyl)imide (**6**) (177 mg, 112 μmol, 1.0 mol eq.), *rac*-BINOL (33.5 mg, 117 μmol, 1.0 mol eq.) and potassium carbonate (13.0 mg, 94.1 μmol, 0.8 mol eq.) were suspended in dry DMF (5 mL) and stirred for 20 h at 130 °C. After cooling down to room temperature, the reaction mixture was poured into a hydrochloric acid solution (2 M, 50 mL). Afterwards, the dark-red solid was filtered and washed with water until a neutral pH-value was obtained and additionally washed with MeOH (50 mL). The solvent was removed via vacuum distillation and the crude product purified by column chromatography (eluent: *n*-hexane/ CH_2Cl_2 = 1/4 v/v %) and recycling GPC ($CHCl_3$) to yield compound **7** as an orange-red solid (64.4 mg, 36.0 μmol, 31 %). **Mp.**: >300 °C. **^{1}H NMR** (400 MHz, CD_2Cl_2): *δ* (ppm) = 10.02 (s, 1H, Ar-*H*), 9.70 (s, 1H, Ar-*H*), 8.87 (s, 1H, Ar-*H*), 8.19 (d, *J* = 8.8 Hz, 1H, Ar-*H*), 8.09 (d, *J* = 8.3 Hz, 1H, Ar-*H*), 8.00 (s, 1H, Ar-*H*), 7.70 (m, 2H, Ar-*H*), 7.62-6.98 (m, 38H, Ar-*H*), 6.76 (d, *J* = 8.4 Hz, 2H, Ar-*H*), 5.72 (d, *J* = 8.9 Hz, 1H, Ar-*H*), 1.10 (m, 27H, Alkyl-C*H*$_3$), 0.99 (m, 27H, Alkyl-C*H*$_3$). **^{13}C NMR** (101 MHz, CD_2Cl_2): *δ* (ppm) = 168.5 (C_q), 168.4 (C_q), 164.1 (C_q), 163.8 (C_q), 163.3 (C_q),

162.8 (C_q), 157.2 (C_q), 154.3 (C_q), 150.9 (C_q), 150.72 (C_q), 150.67 (C_q), 150.6 (C_q), 150.3 (C_q), 149.7 (C_q), 149.4 (C_q), 143.6 (C_q), 143.5 (C_q), 142.2 (C_q), 142.0 (C_q), 141.9 (C_q), 141.6 (C_q), 136.5 (C_q), 136.34 (C_q), 136.28 (C_q), 136.24 (C_q), 136.18 (C_q), 136.1 (C_q), 133.2 (C_q), 132.6 (C_q), 132.0 (C_q), 131.72 (*C*H), 131.67 (*C*H), 131.2 (C_q), 131.1 (*C*H), 131.0 (*C*H), 130.8 (*C*H), 130.7 (*C*H), 130.6 (*C*H), 130.52 (C_q), 130.48 (C_q), 130.3 (*C*H), 129.7 (C_q), 129.1 (C_q), 128.8 (C_q), 128.43, 128.38 (*C*H), 128.3 (*C*H), 128.24 (C_q), 128.15, 128.11 (*C*H), 128.06 (*C*H), 128.0 (*C*H), 127.8 (C_q), 127.6 (C_q), 127.5 (*C*H), 127.3 (C_q), 127.13, 127.10 (*C*H), 126.8 (*C*H), 126.7 (*C*H), 126.2 (*C*H), 125.9 (*C*H), 125.8 (*C*H), 125.7 (*C*H), 125.5 (*C*H), 125.4 (*C*H), 125.3 (*C*H), 125.2 (*C*H), 124.7 (C_q), 124.50 (C_q), 123.9 (C_q), 123.6 (C_q), 123.5 (C_q), 123.5 (C_q), 122.2 (C_q), 121.9 (C_q), 121.6 (C_q), 120.6 (C_q), 120.1 (*C*H), 119.3 (*C*H), 119.0 (C_q), 116.7 (*C*H), 53.8, 34.6 (C_q), 34.6 (C_q), 34.5 (C_q), 31.4 (CH_3), 31.3 (CH_3), 31.2 (CH_3), 31.1 (CH_3). **HRMS** (ESI, positive mode, MeCN/$CHCl_3$): *m/z* calcd for $C_{126}H_{103}N_3NaO_8^+$: [M + Na] $^+$: 1808.76374, found: 1808.77367. **UV/vis** (MCH): λ_{max} = 502 nm (ε_{max} = 30700 M^{-1} cm^{-1}). ***Fluorescence*** (MCH, λ_{ex} = 400 nm): λ_{em} = 513 nm. **QY** (MCH): Φ_{Fl} = (9.4 ± 0.1) %. **Life-time** (MCH): $\tau_{fl,1}$ = 0.6 ns (86 %), $\tau_{fl,2}$ = 1.4 ns (14 %).

The enantiomers of BPTI **7** were separated by semi-preparative HPLC on a chiral stationary phase (eluent: *n*-hexane/CH_2Cl_2 = 2/1 v/v %); flow rate: 6.50 mL min^{-1}; temperature: 22 °C; pressure: 1.20 MPa; detector: UV/vis detector (λ_{mon} = 500 nm). Starting from 8 mg of pure compound *rac*-BPTI **7**, 3.70 mg (46 %) of (*P*)-enantiomer (first elute) and 2.70 mg (34 %) of (*M*)-enantiomer (second elute) were collected. **CD** (MCH) of (*P*)-BPTI **7**: λ_{max} = 401 nm ($\Delta\varepsilon_{max}$ = –24 M^{-1} cm^{-1}). **CD** (MCH) of (*M*)-BPTI **7**: λ_{max} = 401 nm ($\Delta\varepsilon_{max}$ = 25 M^{-1} cm^{-1}). The absolute configuration of the enantiomers was calculated with TDDFT calculations at the wb97xd/6-31g(d) level of theory with the geometry obtained from the crystal structure (Figure S15).

NMR Spectra

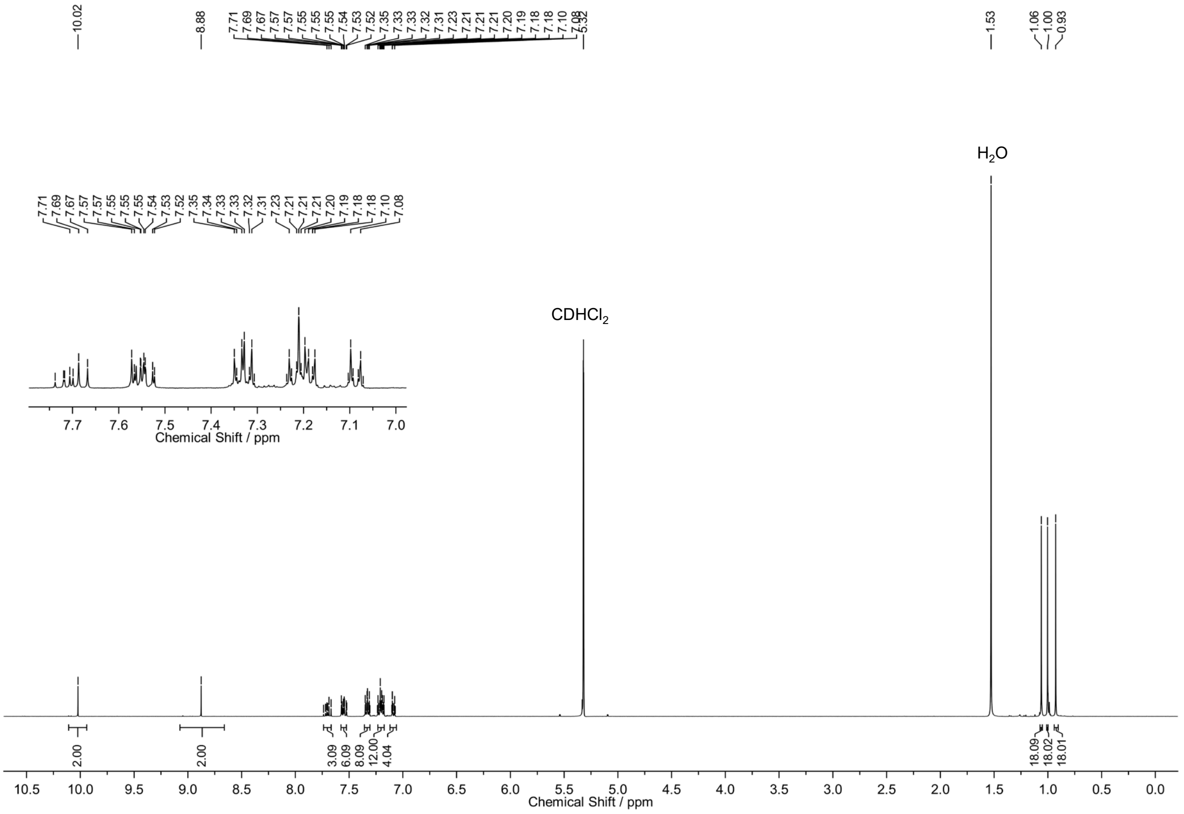

Figure S1. ^{1}H NMR (400 MHz, CD_2Cl_2) spectrum of BPTI **6** at 295 K.

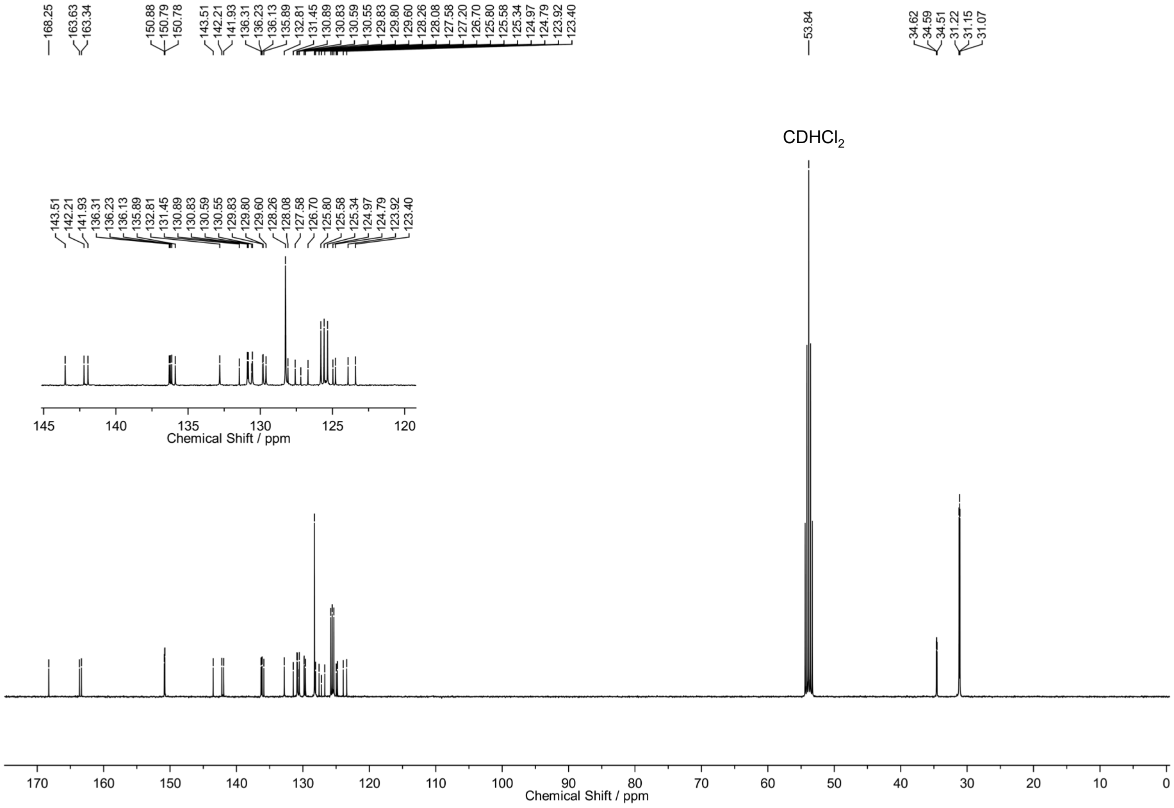

Figure S2. ^{13}C NMR (101 MHz, CD_2Cl_2) spectrum of BPTI **6** at 295 K.

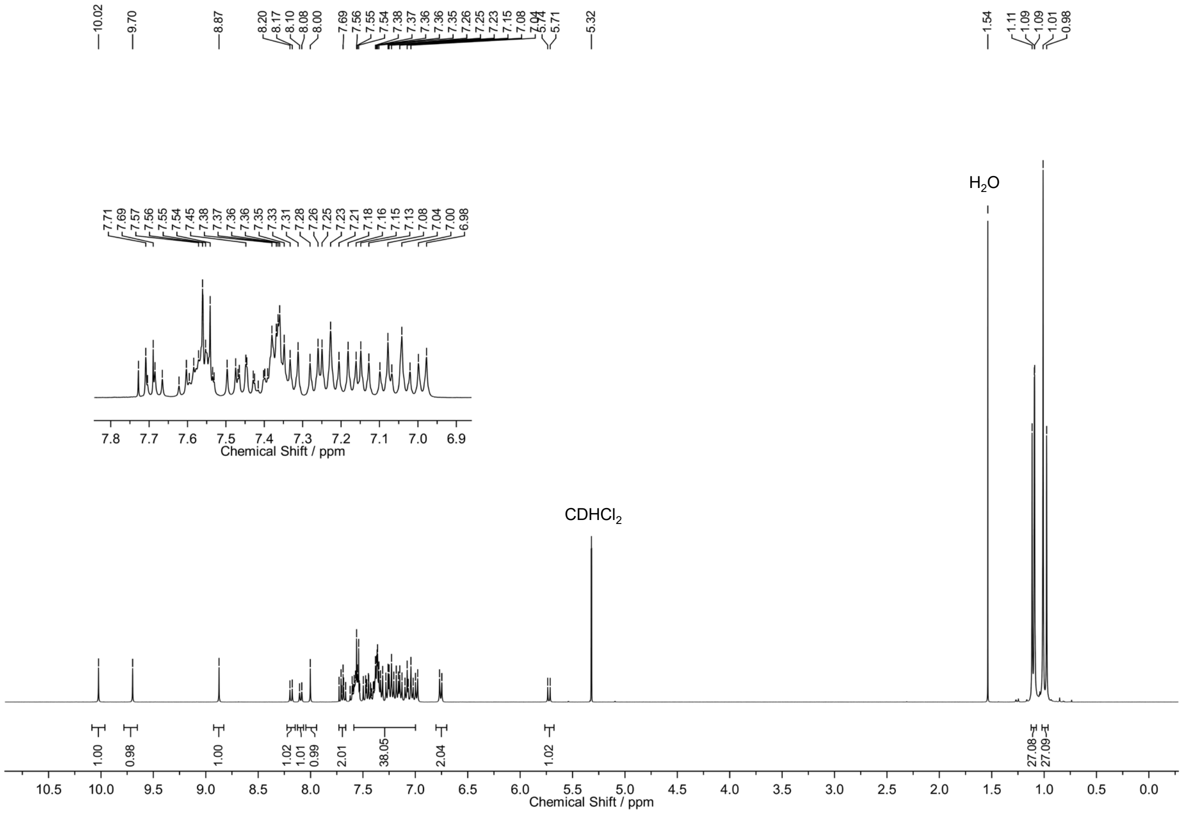

Figure S3. ^{1}H NMR (400 MHz, CD_2Cl_2) spectrum of *rac*-BPTI **7** at 295 K.

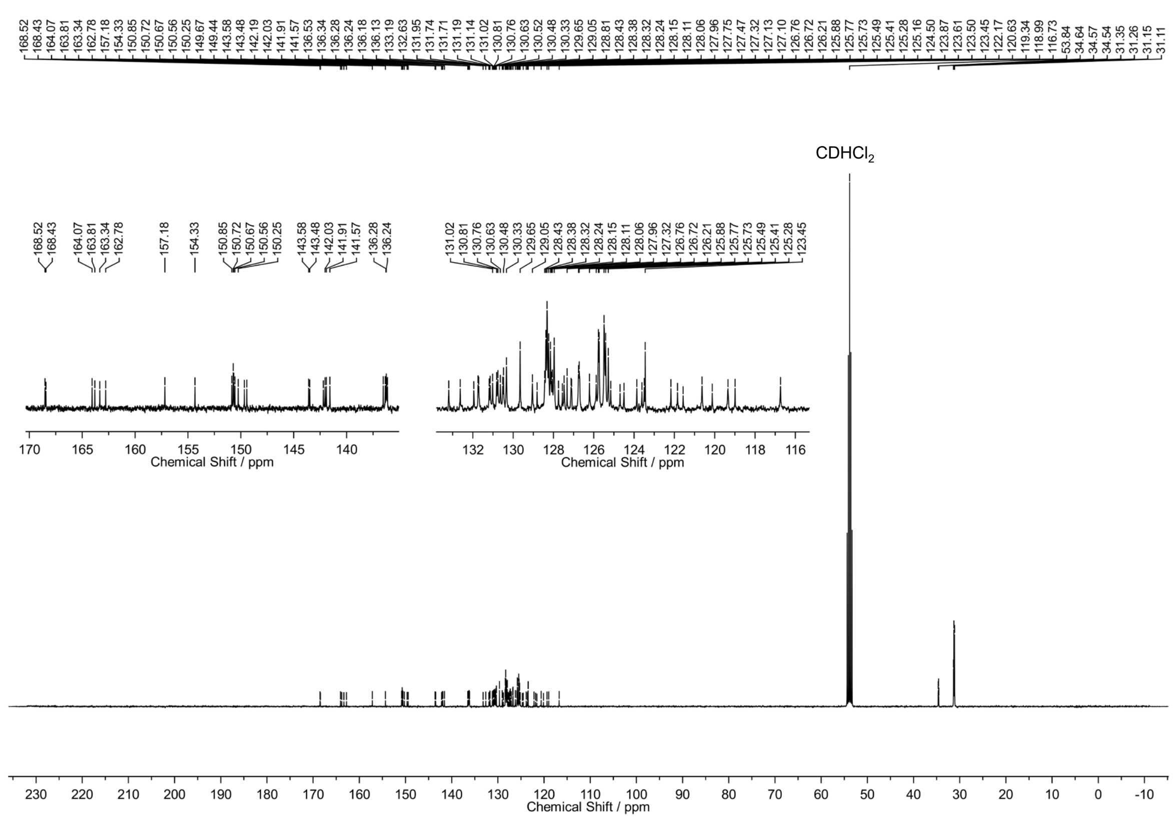

Figure S4. ^{13}C NMR (101 MHz, CD_2Cl_2) spectrum of *rac*-BPTI **7** at 295 K.

Mass Spectra

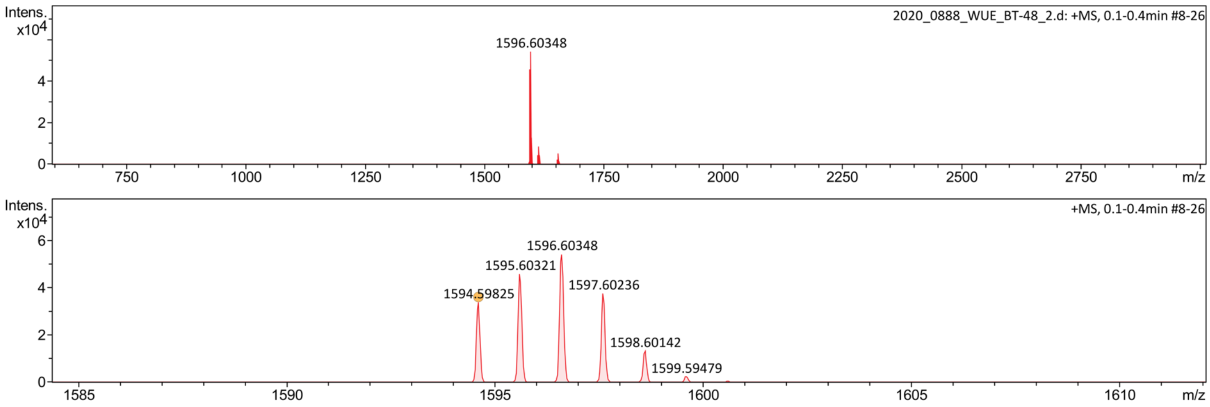

Meas. m/z	#	Ion Formula	m/z	err [ppm]	mSigma	# mSigma	Score	rdb	e⁻ Conf	N-Rule
1594.59825	1	C106H91Cl2N3NaO6	1594.61771	12.21	124.4	1	100.00	61.5	even	ok

Figure S5. HRMS (ESI-TOF, MeCN/$CHCl_3$) of BPTI **6** [M + Na]$^+$.

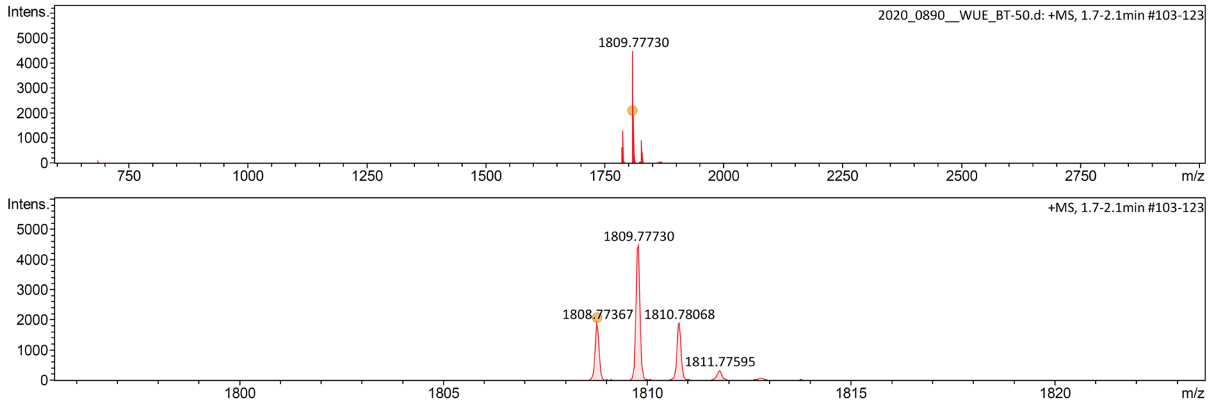

Meas. m/z	#	Ion Formula	m/z	err [ppm]	mSigma	# mSigma	Score	rdb	e⁻ Conf	N-Rule
1808.77367	1	C126H103N3NaO8	1808.76374	-5.49	202.6	1	100.00	76.5	even	ok

Figure S6. HRMS (ESI-TOF, MeCN/$CHCl_3$) of *rac*-BPTI **7** [M + Na]$^+$.

HPLC Separation of the Enantiomers

BPTI **7**:

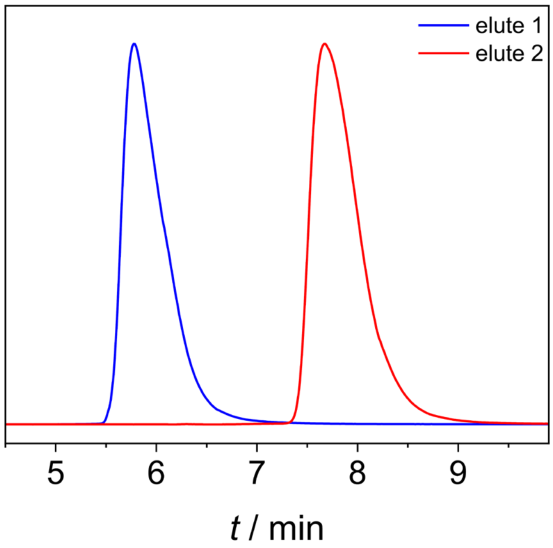

Figure S7. Analytical HPLC of (*P*)-BPTI **7** (blue) and (*M*)-BPTI **7** (red) on a chiral stationary phase (eluent: *n*-hexane/CH_2Cl_2 = 2/1 v/v%); flow rate: 1.0 mL min^{-1}; temperature: 22 °C; pressure: 1.20 MPa; detector: UV/vis detector (λ_{mon} = 510 nm).

[6]helicene (**G5**):

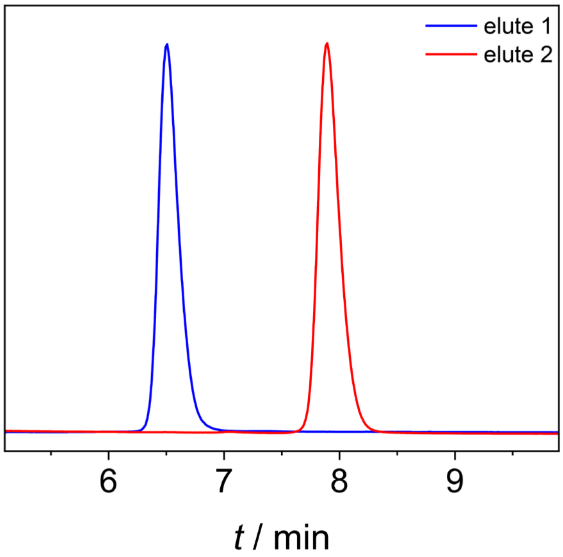

Figure S8. Analytical HPLC of (*P*)-[6]helicene (blue) and (*M*)-[6]helicene (red) on a chiral stationary phase (eluent: *n*-hexane/CH_2Cl_2 = 4/1 v/v%); flow rate: 1.0 mL min^{-1}; temperature: 22 °C; pressure: 1.20 MPa; detector: UV/vis detector (λ_{mon} = 320 nm).

Single Crystal X-ray Analysis

The crystal of *rac*-BPTI **7** was obtained by very slow diffusion (> 6 weeks) of *n*-pentane in a solution of *rac*-BPTI **7** in MeCN and $CHCl_3$.

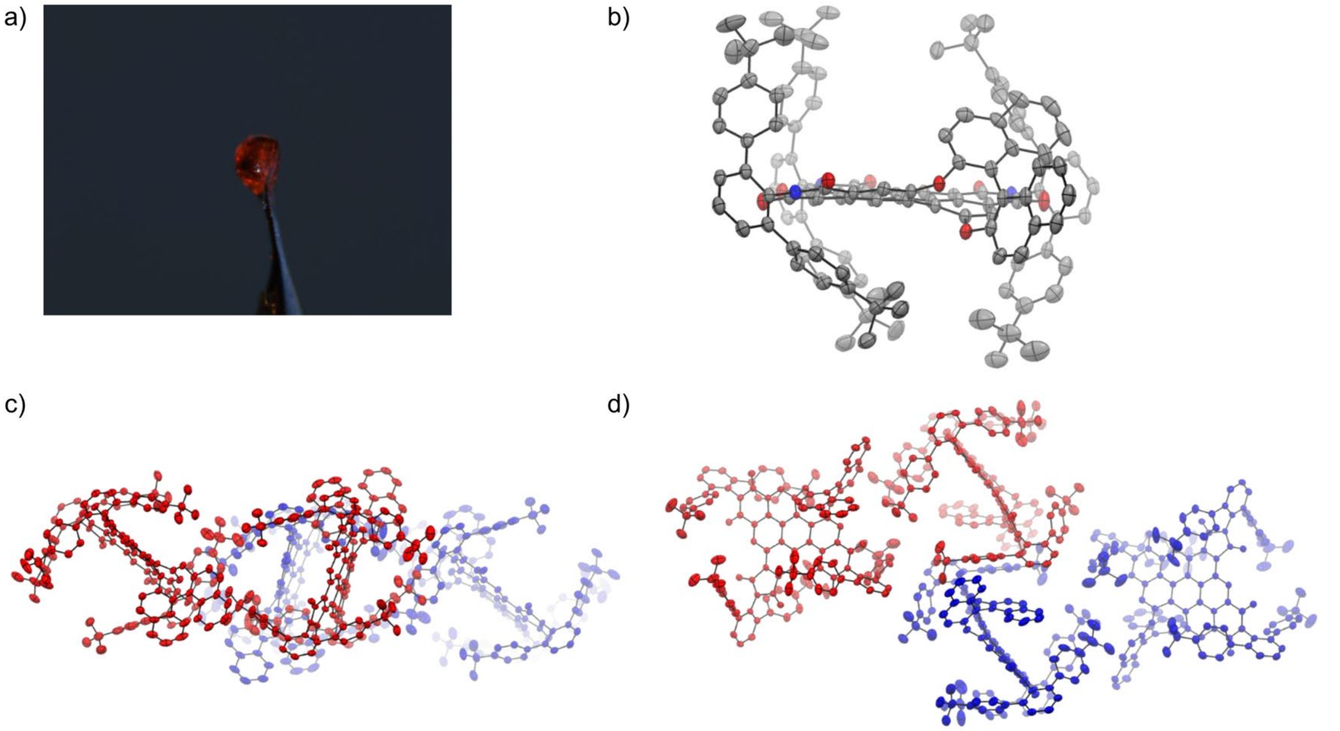

Figure S9. a) Photograph of crystal *rac*-BPTI **7**. b) Side view of molecular structure of BPTI **7** obtained by single-crystal X-ray analysis and two perspectives on the crystal unit cell in c) side view and d) top view with (*P*)- and (*M*)-enantiomers shown in blue and red, respectively. Hydrogen atoms and solvent molecules are omitted for clarity.

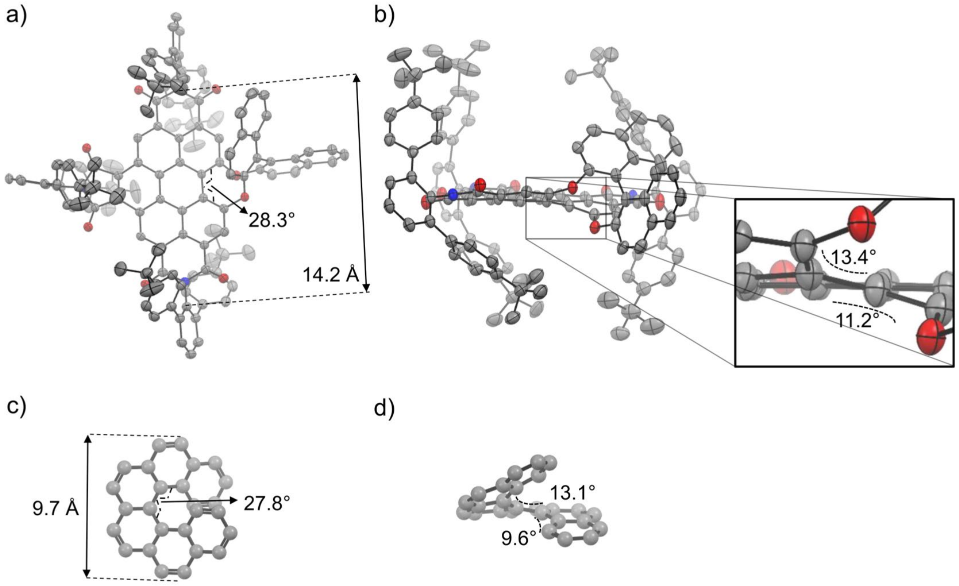

Figure S10. Molecular structure of *rac*-BPTI **7** obtained by single-crystal X-ray analysis. Hydrogen atoms are omitted for clarity in a) top view (distance between the two imide substituents shown, 28.3° as dihedral angle of bay position) b) side view (angles of 13.4° and 11.2° show the alignment of the successive π-planes to each other). and DFT-optimized geometry at the wb97xd/6-31g(d) level of theory of [6]helicene (**G5**) in c) top view (size of 9.7 Å with hydrogen atoms included, 27.8° as dihedral angle of helical planes) and d) side view (angles of 13.1° and 9.6° show the alignment of the successive π-planes to each other).

Table S1. Single crystal X-ray data *rac*-BPTI **7**.

**Compound**	***rac*-BPTI 7**
**CCDC Number**	2132523
**Sum formula**	$C_{130}H_{109}N_5O_8$
**Molecular weight (g·mol^{-1})**	1869.22
**Crystal size**	0.251 x 0.167 x 0.120 mm
**Temperature / K**	100 (2)
**Wavelength / Å**	1.54178
**Crystal colour**	red
**Crystal system**	monoclinic
**Space group**	P2 (1)
**a / Å**	13.8382 (5)
**b / Å**	30.2689 (11)
**c / Å**	26.5850 (9)
**α / °**	90
**β / °**	92.719 (2)
**γ / °**	90
**Volume (Å^3)**	11123.0 (7)
**Z**	4
**Calculated density / g cm^{-3}**	1.116
**Absorption coefficient / mm^{-1}**	0.541
**F(000)**	3952
**Measurement range of *θ* / °**	2.213 to 72.603
**Limiting indices**	–17 ≤ h ≤ 17, –37 ≤ k ≤ 37, –27 ≤ 1 ≤ 32
**Reflections collected / unique**	186320 / 22047 [R (int) = 0.0625]
**Completeness / %**	100 %
**Absorption correction**	semi-empirical from equivalents
**T_{min}, T_{max}**	0.7022, 0.7536
**Refinement method**	full-matrix least-squares on F^2
**Data / restraints / parameters**	22047 / 323 / 1365
**Goodness-of-fit on F^2**	1.038
***R* [I > 2σ(I)]**	R_1 = 0.0586, wR_2 = 0.1511
***R* (all data)**	R_1 = 0.0693, wR_2 = 0.1598
**Largest diff. peak and hole**	0.489 and –0.424 e.Å^{-3}

Activation Parameters for the Racemization of BPTI 7

To determine the activation parameters of BPTI **7** the racemization was monitored by time-dependent CD spectroscopy at different temperatures. Because of the high activation barrier, temperatures of 473-523 K were chosen and therefore the high boiling diphenylether was used as solvent. All samples were prepared in a temperature-resistant vial and heated to the respective temperature with an oil bath that has already been heated to the desired temperature. For each measurement, the solution was rapidly cooled with cold water and then a CD spectrum recorded. Afterwards, the vial with solution was again placed in the oil bath with the respective temperature. An uncertainty for the temperature of $\sigma T = \pm 5$ K was assumed. The timer for racemization was started each time before heating and stopped before cooling the sample. It was assumed that no racemization takes place during the CD measurement at 293 K.

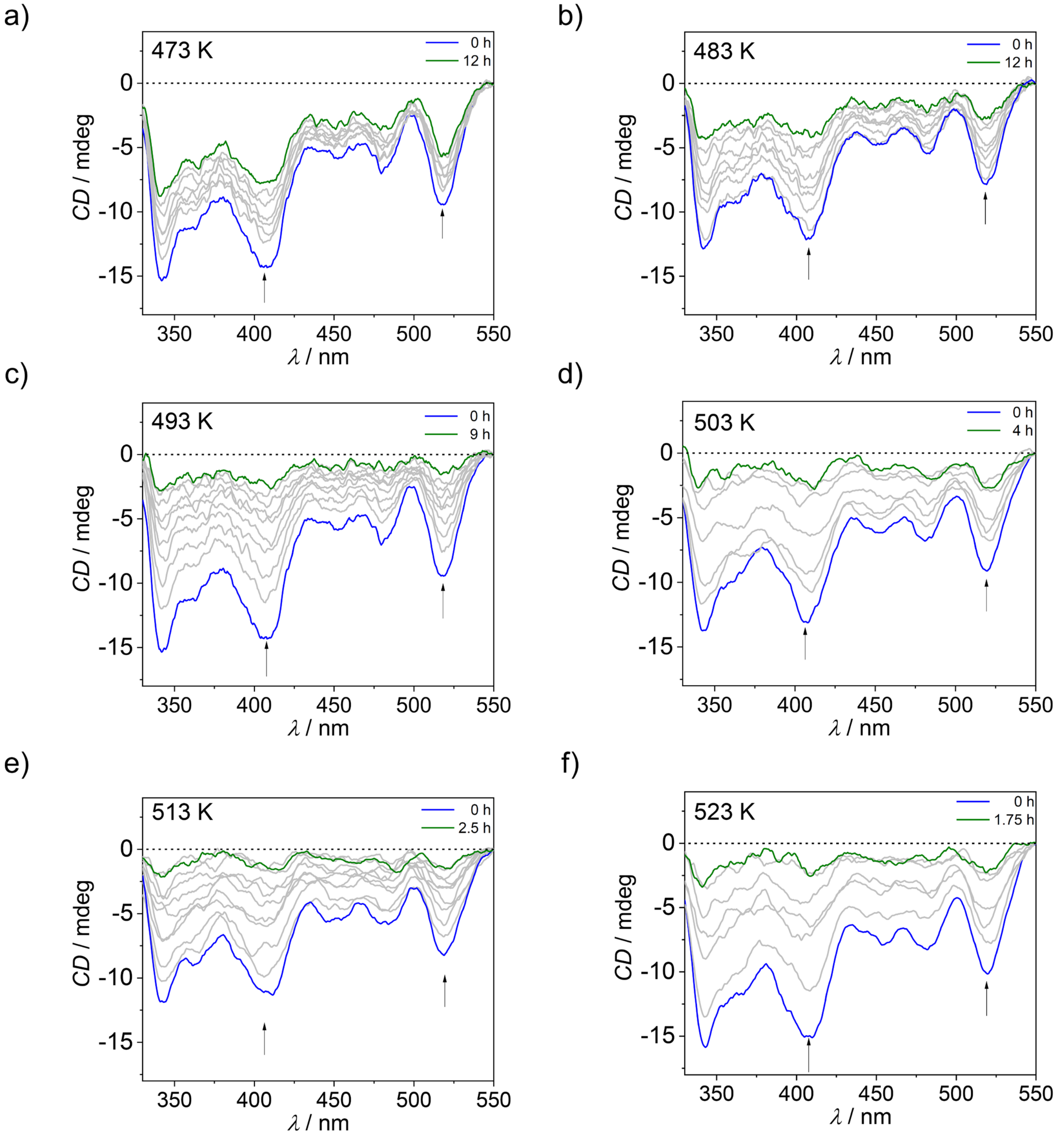

Figure S11. Racemization of (*P*)-BPTI **7** in diphenylether at a) 473 K b) 483 K c) 493 K d) 503 K e) 513 K and f) 523 K monitored by time dependent CD spectroscopy.

Racemization follows a first order kinetic, so that the rate law for the racemization between (*P*)- and (*M*) enantiomer is given by:

$$v = -\frac{d(A)}{A_0} = k\,dt \qquad \text{(S1)}$$

Where A is the concentration of the enantiomer present in excess and A_0 is the initial concentration of this enantiomer. Due to the linear relationship between CD amplitude and the enantiomer concentration, A/A_0 was calculated globally from the integrals (325-550 nm) of the time-dependent CD spectra (Figure S11).

$$A = A_0 e^{-kt} \rightarrow \ln\frac{A}{A_0} = -kt \qquad y(x) = \mathrm{m}x + \mathrm{c} \qquad \text{(S2)}$$

$$\mathrm{m} = -k \;\;\&\;\; \mathrm{c} = 0 \qquad \text{(S3)}$$

The rate constant k at certain temperatures can be determined by plotting the ln of A/A_0 versus the time, which gives $-k$ as the slope of the linear fit of the data points (Figure S12).

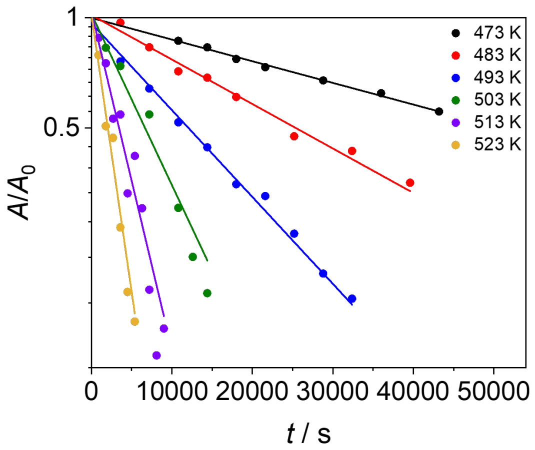

Figure S12. The logarithmic plot of A/A_0 versus time shows the racemization rates of (*P*)-BPTI **7** in diphenylether at 473 K (black), 483 K (red), 493 K (blue), 503 K (green), 513 K (purple) and 523 K (yellow).

The racemization rate allows to calculate the half-life $t_{½}$:

$$t_{1/2} = \frac{\ln 2}{k} \qquad \text{(S4)}$$

With the Eyring equation (S5) the rate constant is related at different temperatures to the racemization barrier $\Delta G^{‡}$:

$$\Delta G^{‡} = -RT\ln\frac{kh}{\kappa k_B T} \qquad \text{(S5)}$$

Where κ is a transmission coefficient and for the racemization process $\kappa = 0.5$ since the probability of the transition state to transform into the one or the other enantiomer is equal.[S8] h is Planck´s constant (6.63 × 10^{-34} J s), k_B the Boltzmann constant (1.38 × 10^{-23} J K^{-1}), T the temperature and R the gas constant (8.314 J mol^{-1} K^{-1}). By rearranging the Eyring equation (S6) $\Delta G^{‡}$ as well as the enthalpy $\Delta H^{‡}$ and the entropy $\Delta S^{‡}$ can be determined by plotting ln (k/T) versus $1/T$ (Figure S13a):

$$\ln\frac{k}{T} = -\left(\frac{\Delta H^{‡}}{R}\right)\frac{1}{T} + \frac{\Delta S^{‡}}{R} + \ln\frac{\kappa k_B}{h} \qquad y(x) = \mathrm{m}x + \mathrm{c} \qquad \text{(S6)}$$

$$\mathrm{m} = -\left(\frac{\Delta H^{‡}}{R}\right) \;\;\&\;\; \mathrm{c} = \frac{\Delta S^{‡}}{R} + \ln\frac{\kappa k_B}{h} \qquad \text{(S7)}$$

Likewise, the activation energy E_A can be determined according to Arrhenius (S8) by plotting ln(k) versus $1/T$ with ($-E_A/R$) as the slope and ln(A) as the y axis intercept of the plot (Figure S13b).

$$k = Ae^{-\frac{E_A}{RT}} \rightarrow \ln k = -\left(\frac{E_A}{R}\right)\frac{1}{T} + \ln A \qquad y(x) = \mathrm{m}x + \mathrm{c} \qquad \text{(S8)}$$

$$\mathrm{m} = -\left(\frac{E_A}{R}\right) \;\;\&\;\; \mathrm{c} = \ln A \qquad \text{(S9)}$$

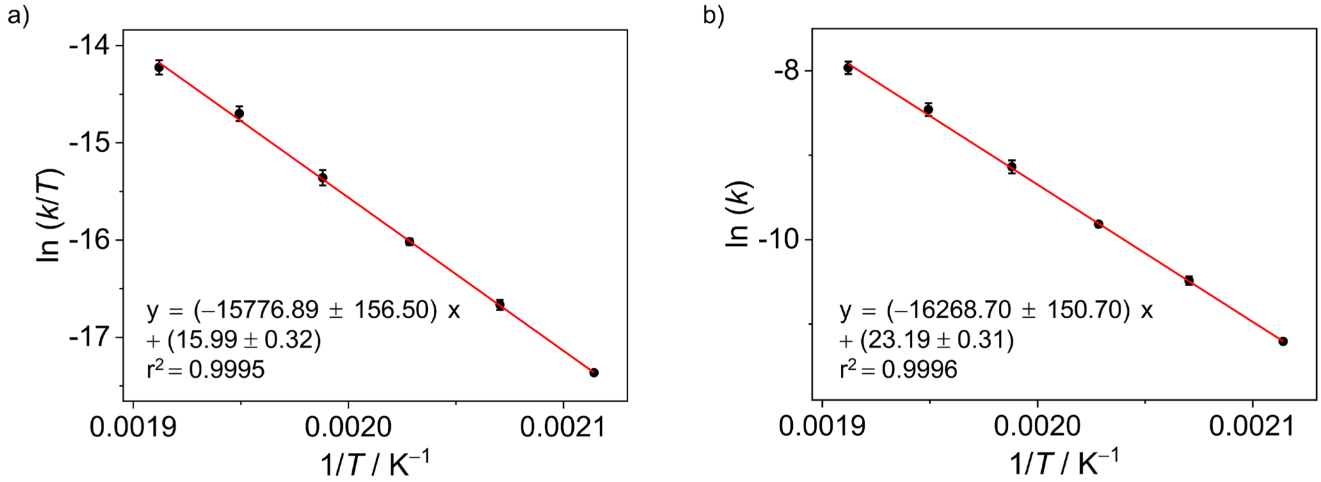

Figure S13. a) Eyring plot ln(*k*/*T*) versus 1/*T* for (*P*)-BPTI **7** at 473 K, 483 K, 493 K, 503 K, 513 K and 523 K. b) Arrhenius plot ln(*k*) versus 1/*T* for the racemization of (*P*)-BPTI **7** at 473 K, 483 K, 493 K, 503 K, 513 K and 523 K.

The uncertainty σ of all values were determined by the Gaussian error propagation law (S10). E.g. $\sigma\Delta G^{\ddagger}$ was calculated via equation (S11).

$$\sigma_y = \sqrt{(\sigma_1 \frac{\partial y}{\partial x_1})^2 + (\sigma_2 \frac{\partial y}{\partial x_2})^2 + \ldots} \qquad \text{(S10)}$$

$$\sigma\Delta G^{\ddagger} = \sqrt{(\sigma k \left| -\frac{RT}{k} \right|)^2 + (\sigma T \left| R - R\ln\frac{hk}{\kappa k_B T} \right|)^2} \qquad \text{(S11)}$$

Table S2. Summary of activation parameters for racemization of (*P*)-BPTI **7**, in diphenylether.

**Analyte**	**$\Delta G^{\ddagger}$ [kJ mol^{-1}]**[a,b]	**$\Delta H^{\ddagger}$ [kJ mol^{-1}]**[c,d]	**$\Delta S^{\ddagger}$ [J mol^{-1} K^{-1}]**[c,d]	**E_A [kJ mol^{-1}]**[e,f]	**T [K]**	**k [s^{-1}]**[g,h]	**$t_{1/2}$ [h]**[h,i]
(*P*)-BPTI **7**	159 ± 2	132 ± 1	–59 ± 3	135 ± 1	473	(1.36 ± 0.03) 10^{-5}	14.16 ± 0.32
	160 ± 2				483	(2.79 ± 0.14) 10^{-5}	6.90 ± 0.35
	160 ± 2				493	(5.45 ± 0.18) 10^{-5}	3.53 ± 0.11
	161 ± 2				503	(1.07 ± 0.08) 10^{-4}	1.79 ± 0.14
	161 ± 2				513	(2.12 ± 0.16) 10^{-4}	0.91 ± 0.07
	162 ± 2				523	(3.48 ± 0.26) 10^{-4}	0.55 ± 0.04

[a] Calculated with the Eyring equation (S5) at respective temperatures. [b] Error calculated from the error of the linear regression (Figure S12) and σT = ±5 K and is calculated with the equation (S11). [c] Calculated with the Eyring plot (Figure S13a) and equation (S7). [d] Error calculated from the error of the linear regression (Figure S13a). [e] Calculated with the Arrhenius plot (Figure S13b) and equation (S9). [f] Error calculated from the error of the linear regression (Figure S13b). [g] Calculated with temperature dependent CD spectroscopy (Figure S11) and an exponential fit via equation (S2). [h] For the error of *k*, the error of the exponential fit (Figure S12) was used. [i] Calculated with the equation (S4).

Optical Spectroscopy

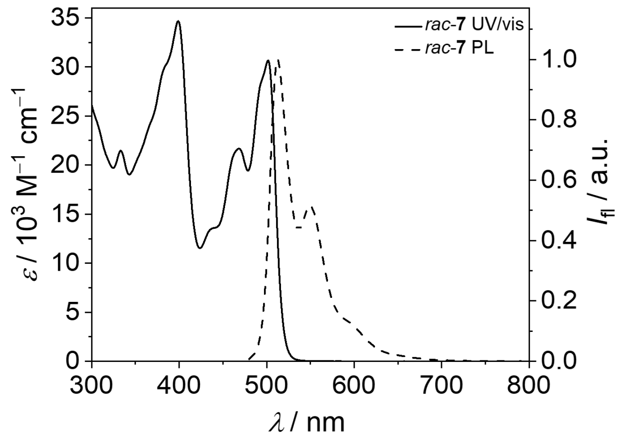

Figure S14. UV/vis absorption spectrum of *rac*-BPTI **7** (black solid line; c = 1.1 × 10^{-5} M) and normalized emission spectrum of *rac*-BPTI **7** (black dashed line; c ~ 10^{-7} M, λ_{ex} = 400 nm) in MCH at 293 K.

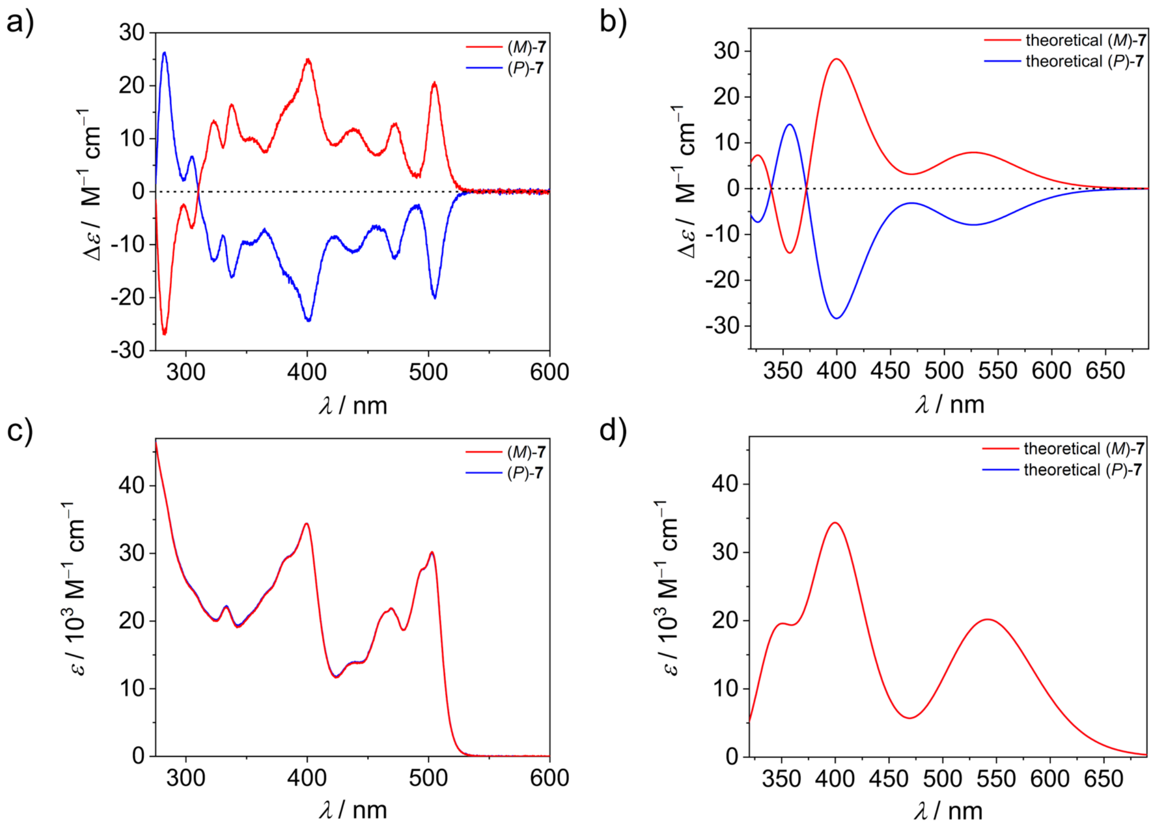

Figure S15. a) CD (c ~ 10^{-5} M) at 293 K, b) theoretical CD (DFT-optimized TDDFT calculation at the wb97xd/6-31g(d) level of theory), c) UV/vis absorption (c ~ 10^{-5} M) at 293 K and d) theoretical UV/vis (DFT-optimized TDDFT calculation at the wb97xd/6-31g(d) level of theory) spectra of (*M*)-BPTI **7** (red line) and (*P*)-BPTI **7** (blue line) in MCH.

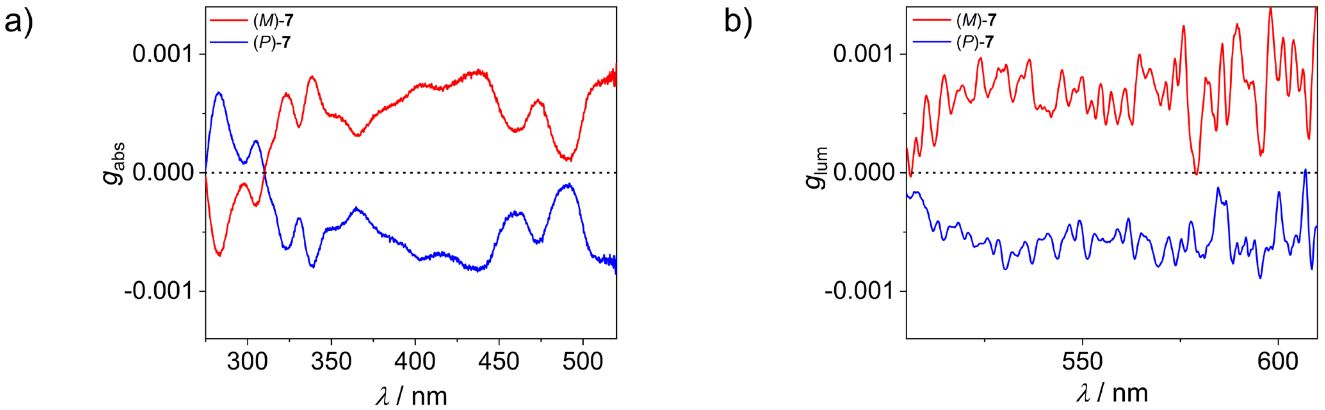

Figure S16. Wavelength dependent a) g_{abs} and b) g_{lum} of (*M*)-BPTI **7** (red line; c = 3.87 × 10^{-5} M; λ_{ex} = 400 nm) and (*P*)-BPTI **7** (blue line; c = 3.75 × 10^{-5} M; λ_{ex} = 400 nm) in MCH at 293 K.

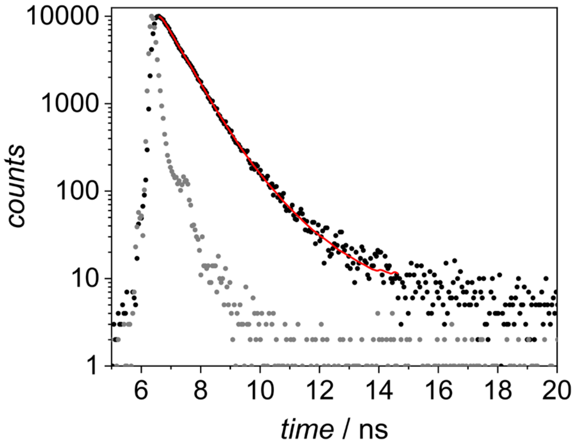

Figure S17. Life-time measurement (black) of *rac*-BPTI **7** (MCH, 293 K, λ_{ex} = 404 nm, λ_{em} = 513 nm) and IRF (grey). The biexponential fit is shown in red.

Table S3. Optical properties of BPTI **7** in MCH solution determined by UV/vis, CD, fluorescence spectroscopy and CPL.

BPTI 7	MCH
λ_{abs} (S_1, $A_{0\text{-}0}$) [nm]	502
λ_{abs} (S_1, $A_{0\text{-}1}$) [nm]	468
λ_{abs} (S_2, $A_{0\text{-}0}$) [nm]	400
ε_{max} (S_1, $A_{0\text{-}0}$) [M^{-1} cm^{-1}]	30700
ε_{max} (S_1, $A_{0\text{-}1}$) [M^{-1} cm^{-1}]	21700
ε_{max} (S_2, $A_{0\text{-}0}$) [M^{-1} cm^{-1}]	34500
$\Delta\varepsilon_{max}$ (*M*)[a] [M^{-1} cm^{-1}]	25
$\Delta\varepsilon_{max}$ (*P*)[b] [M^{-1} cm^{-1}]	–24
λ_{em} [nm][c]	513
$\Delta\tilde{\nu}_{Stokes}$ [cm^{-1}][d]	427
Φ_{Fl} [%][e]	9.4 ± 0.1
$\tau_{Fl,1}$ [ns][f]	0.6
(ampl./%)[f]	(86)
$\tau_{Fl,2}$ [ns][f]	1.4
(ampl./%)[f]	(14)
k_r [10^8 s^{-1}][g]	1.32
k_{nr} [10^9 s^{-1}][g]	1.27
g_{abs}[h]	0.0007
g_{lum}[i]	0.0010
B_{CPL}[j] [M^{-1} cm^{-1}]	1.62

[a] $\Delta\varepsilon_{max}$ at 401 nm of (*M*)-BPTI **7**. [b] $\Delta\varepsilon_{max}$ at 401 nm of (*P*)-BPTI **7**. [c] λ_{ex} = 400 nm. [d] Stokes shift between λ_{abs} (S_1, $A_{0\text{-}0}$) and λ_{em}. [e] The fluorescence quantum yield of BPTI **7** was measured relative to *N*,*N'*-bis(2,6-diisopropylphenyl)-3,4:9,10-bis(dicarboxdiimide) (Φ_{Fl} = 100 % in $CHCl_3$)[S6] as reference at four different excitation wavelengths. [f] For life-time measurements a pulsed laser diode with a wavelength of 403.8 nm was used. [g] Determined according to $k_r = \Phi_{Fl}/\tau_{Fl}$ and $k_{nr} = 1/\tau_{Fl} - k_r$. [h] g_{abs} at 400 nm of (*M*)-BPTI **7**. [i] g_{lum} at 524 nm of (*M*)-BPTI **7**. [j] Determined according to $B_{CPL} = \lambda_{abs}\Phi_{Fl}|g_{lum}|/2$.[S9]

(Spectro-) Electrochemical Properties

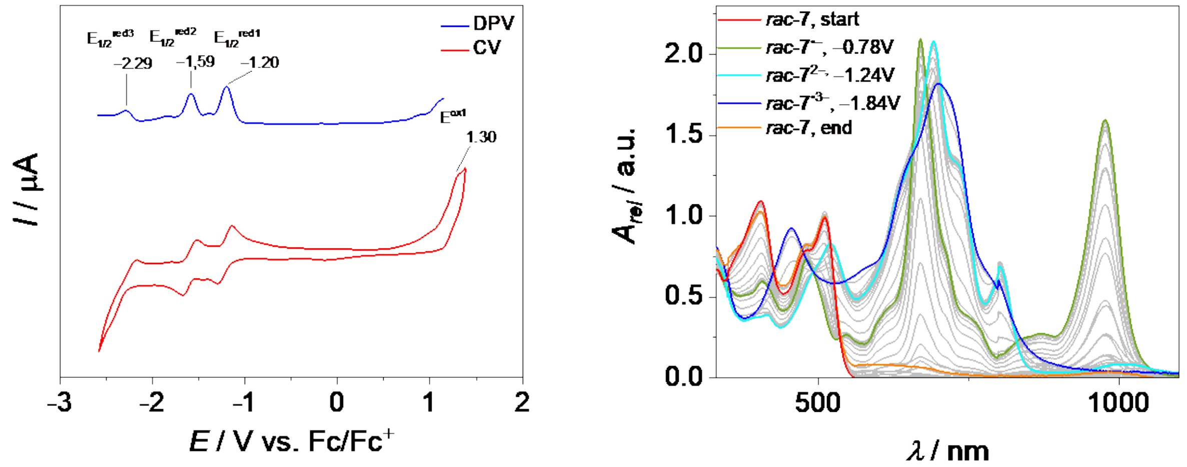

Figure S18. a) Differential pulse voltammogram (blue line) and cyclic voltammogram (red line) of *rac*-BPTI **7** ($c \sim 2.50 \times 10^{-4}$ M) in CH_2Cl_2 containing tetrabutylammonium hexafluorophosphate ($c \sim 1.00 \times 10^{-2}$ M) as a supporting electrolyte versus Fc/Fc^{+} at a scan rate of 100 mV s^{-1} and 293 K. b) UV/vis spectral changes in the first (green), second (cyan) and third electron reduction (blue) of *rac*-BPTI **7** in deaerated DMF that contained NBu_4PF_6 (0.1 M) at 293 K.

Titration Studies

For the titration experiments a solution of enantiomerically pure or racemic BPTI **7** and the respective guest in excess was titrated to a solution of the pure BPTI **7** solution by keeping the concentration of BPTI **7** constant. As the titrations were all carried out in MCH the concentration of the guest solution was limited by solubility. Theoretical spectra (black dotted lines) for each titration end point (corresponding to 1:1 complex) were calculated by using the following equations:

$$A_{rel} = x_{HG}A_{HG} + x_{H}A_{H} \qquad \text{(S12)}$$

$$A_{HG} = \frac{A_{rel} - x_{H}A_{H}}{x_{HG}} \qquad \text{(S13)}$$

With A_H as the absorbance of the host molecule (BPTI **7**), A_{HG} as the absorbance of the host-guest complex and A_{rel} as the measured absorption of a mixture of host and host-guest complex. The coefficients x_H and x_{HG} represent the mole fraction of the host and the host-guest complex, respectively. Both mole fractions were calculated from the experimental data via the global 1:1 fit of *bindfit*.[S10]

The respective binding constants K_a were calculated by a 1:1 nonlinear curve fit for specific wavelengths and a global 1:1 fit via the program *bindfit*.[S10] Since the values obtained were by both approaches within the expected error range, the binding constants of the global fit were used in the discussion in the main section. For the studies using racemic mixtures of the host and [4]- to [6]helicene it was assumed, that only the respective homochiral complex will form in solution, so for the calculation of K_a the concentration of host and guest was divided by two.

UV/vis Titrations

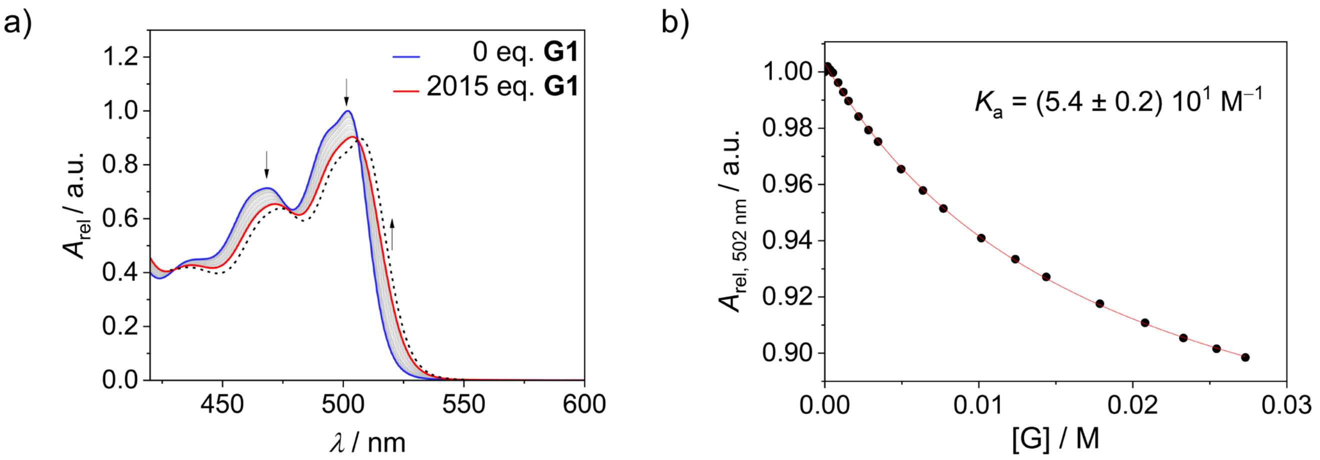

Figure S19. UV/vis spectra of *rac*-BPTI **7** in MCH at 293 K (c = 1.4 x 10^{-5} M) upon the addition of naphthalene (**G1**) as a guest and b) the resulting plot of the absorption changes at λ = 502 nm with nonlinear curve fit (1:1 binding model, red curve).

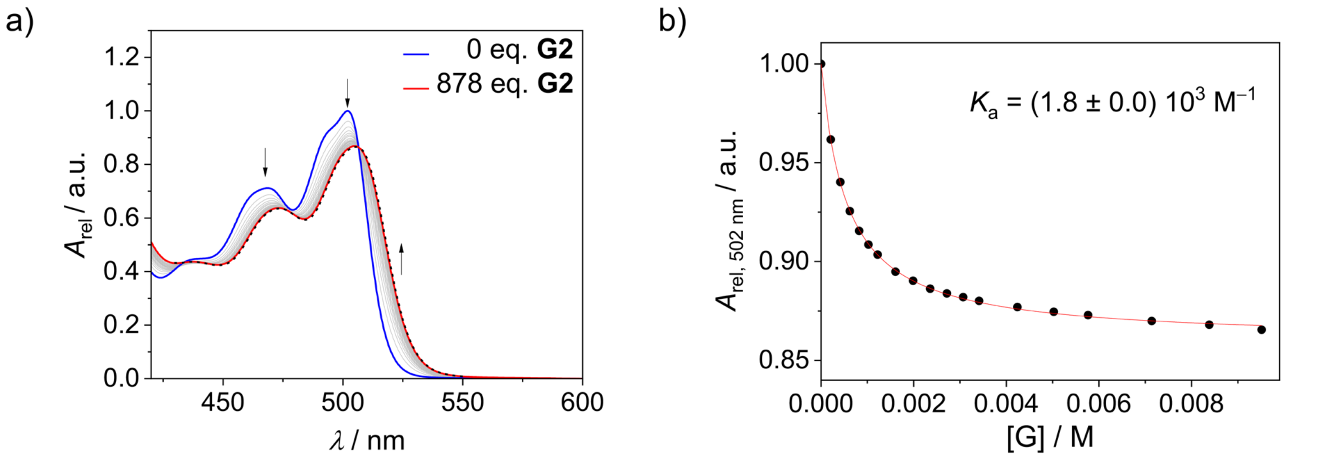

Figure S20. UV/vis spectra of *rac*-BPTI **7** in MCH at 293 K (c = 1.7 x 10^{-5} M) upon the addition of phenanthrene (**G2**) as a guest and b) the resulting plot of the absorption at λ = 502 nm with nonlinear curve fit (1:1 binding model, red curve).

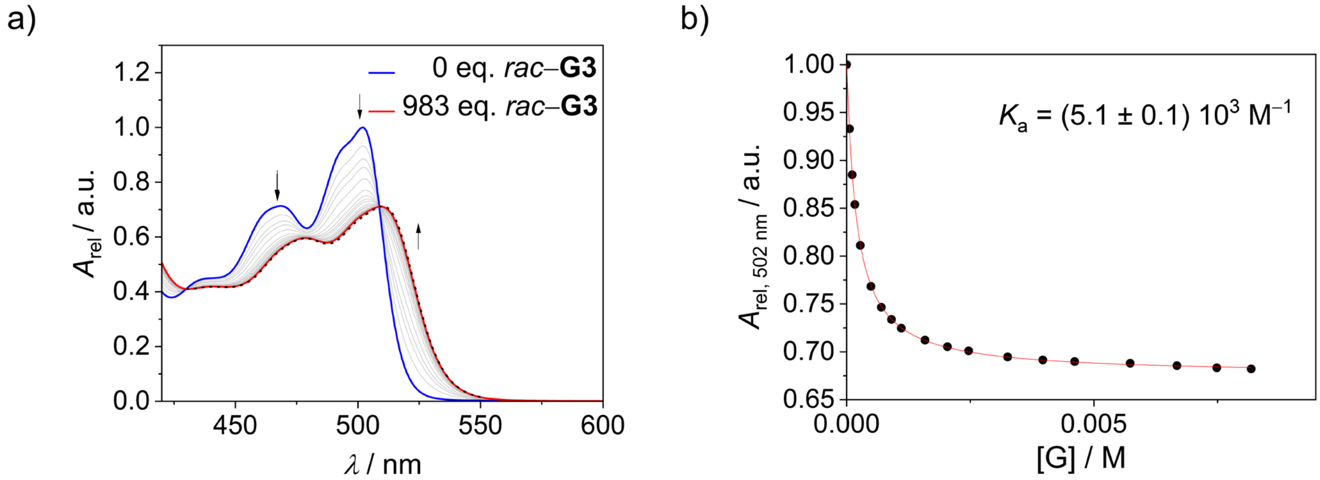

Figure S21. UV/vis spectra of *rac*-BPTI **7** in MCH at 293 K (c = 1.7 x 10^{-5} M) upon the addition of *rac*-[4]helicene (**G3**) as a guest and b) the resulting plot of the absorption at λ = 502 nm with nonlinear curve fit (1:1 binding model, red curve).

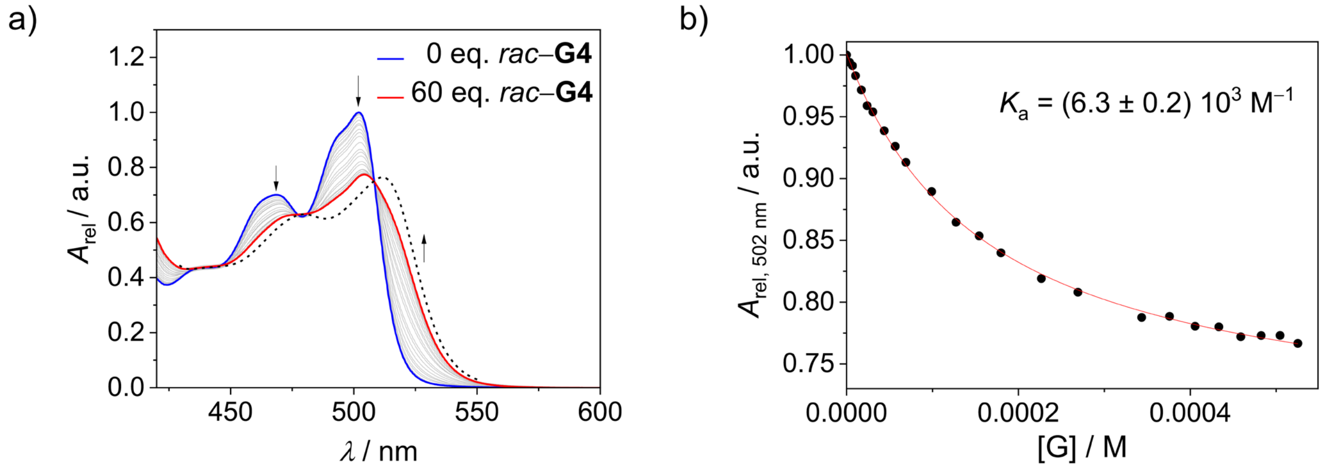

Figure S22. UV/vis spectra of *rac*-BPTI **7** in MCH at 293 K (c = 1.7 x 10^{-5} M) upon the addition of *rac*-[5]helicene (**G4**) as a guest and b) the resulting plot of the absorption at λ = 502 nm with nonlinear curve fit (1:1 binding model, red curve).

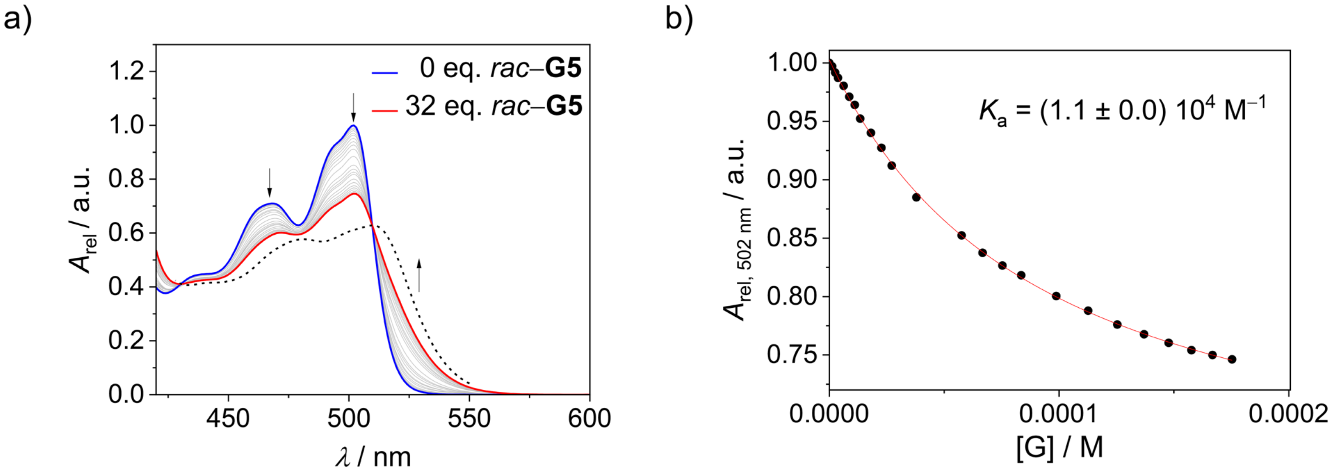

Figure S23. UV/vis spectra of *rac*-BPTI **7** in MCH at 293 K (c = 1.1 x 10^{-5} M) upon the addition of *rac*-[6]helicene (**G5**) as a guest and b) the resulting plot of the absorption at λ = 502 nm with nonlinear curve fit (1:1 binding model, red curve).

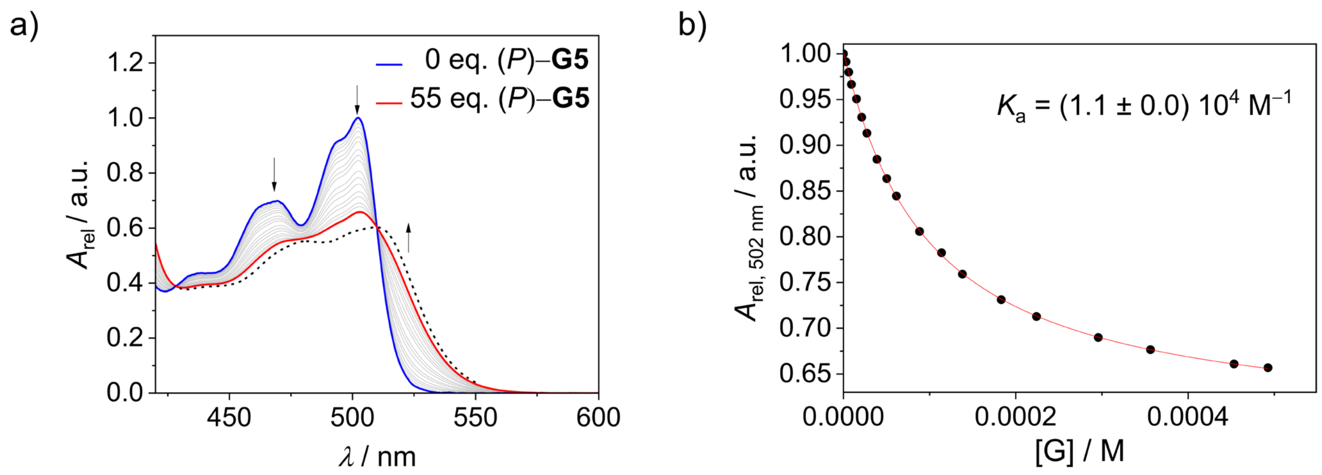

Figure S24. UV/vis spectra of (*P*)-BPTI **7** in MCH at 293 K (c = 9.0 x 10^{-6} M) upon the addition of (*P*)-[6]helicene (**G5**) as a guest and b) the resulting plot of the absorption at λ = 502 nm with nonlinear curve fit (1:1 binding model, red curve).

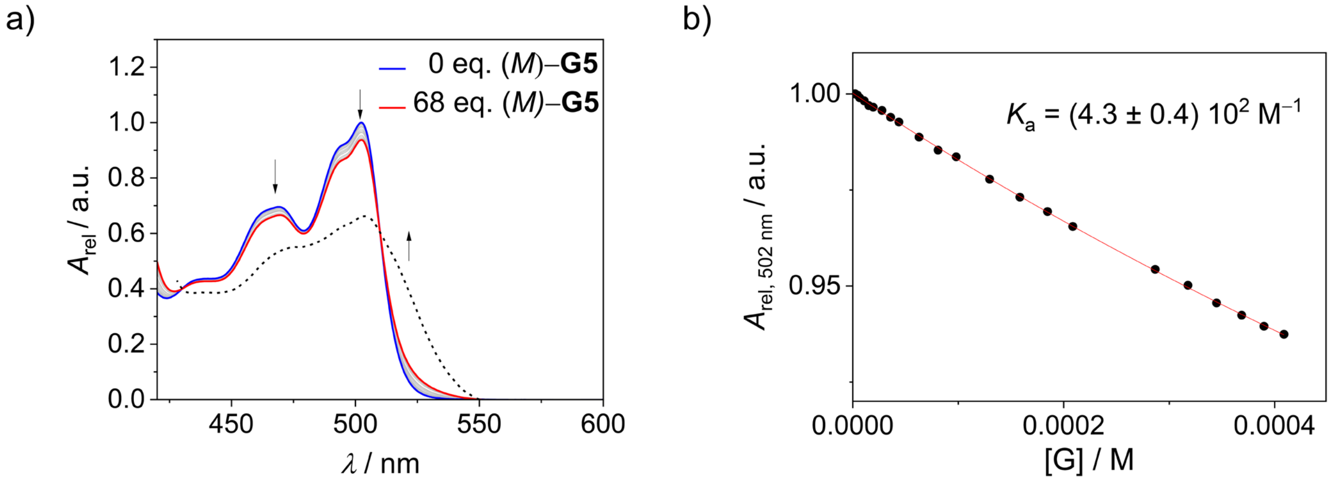

Figure S25. UV/vis spectra of (*P*)-BPTI **7** in MCH at 293 K (c = 6.0 x 10^{-6} M) upon the addition of (*M*)-[6]helicene (**G5**) as a guest and b) the resulting plot of the absorption at λ = 502 nm with nonlinear curve fit (1:1 binding model, red curve).

Fluorescence Titrations

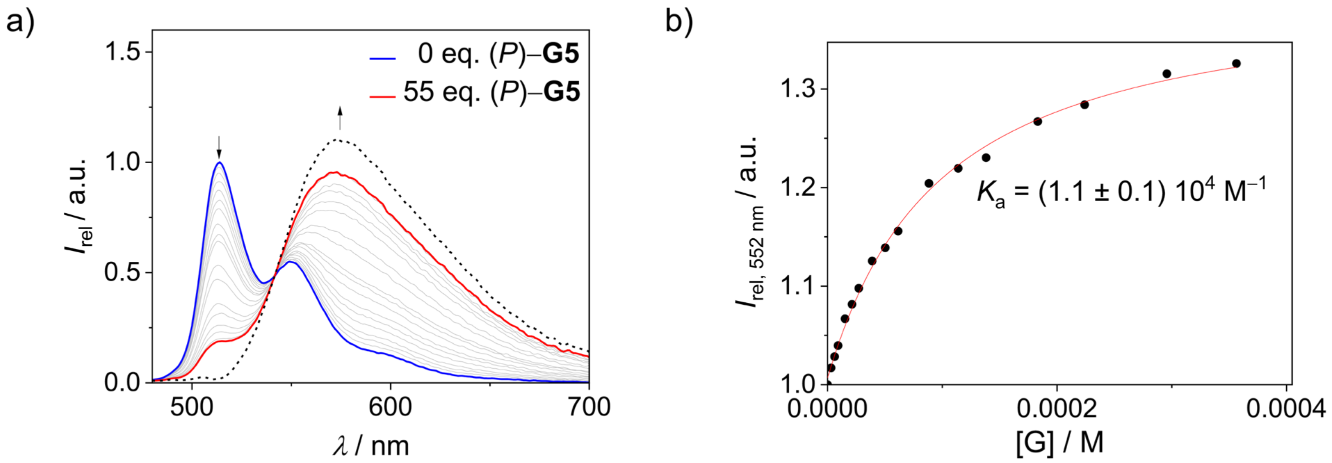

Figure S26. a) Fluorescence spectra of (*P*)-BPTI **7** in MCH at 293 K (λ_{ex} = 428 nm, *c* = 9.0 x 10^{-6} M) upon the addition of (*P*)-[6]helicene (**G5**) as a guest and b) the resulting plot of the emission at λ = 552 nm with nonlinear curve fit (1:1 binding model, red curve).

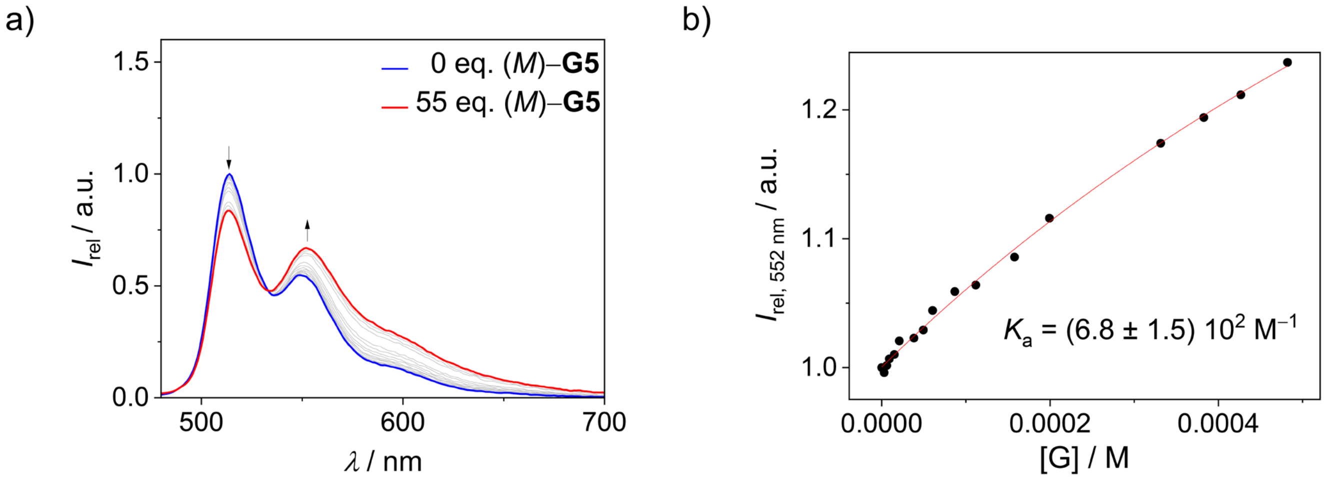

Figure S27. a) Fluorescence spectra of (*P*)-BPTI **7** in MCH at 293 K (λ_{ex} = 428 nm, *c* = 8.7 x 10^{-6} M) upon the addition of (*M*)-[6]helicene (**G5**) as a guest and b) the resulting plot of the emission at λ = 552 nm with nonlinear curve fit (1:1 binding model, red curve).

CD titrations

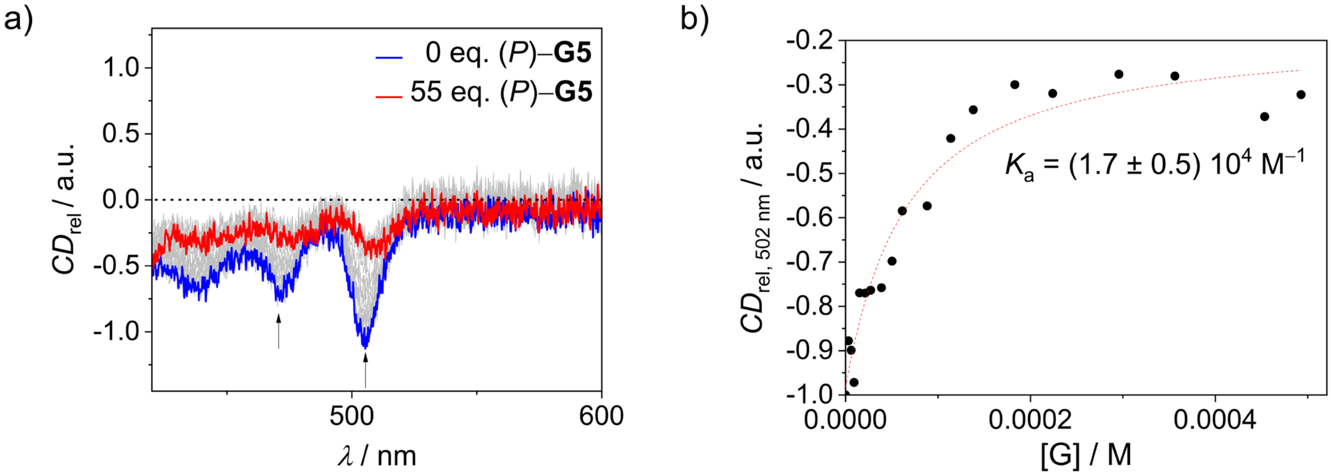

Figure S28. a) CD spectrum of *rac*-BPTI **7** in MCH at 293 K (λ_{ex} = 428 nm, c = 9.0 x 10^{-6} M) upon the addition of (*P*)-[6]helicene (**G5**) as a guest and b) the resulting plot of the absorption at λ = 502 nm with nonlinear curve fit (1:1 binding model, red curve).

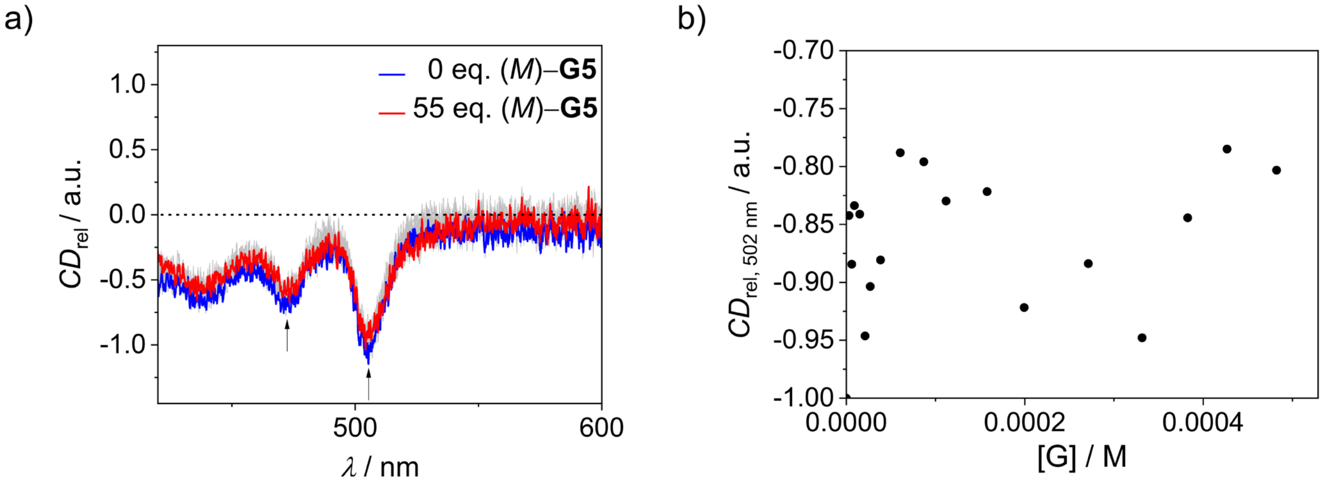

Figure S29. a) CD spectrum of *rac*-BPTI **7** in MCH at 293 K (λ_{ex} = 428 nm, c = 8.7 x 10^{-6} M) upon the addition of (*M*)-[6]helicene (**G5**) as a guest and b) the resulting plot of the absorption at λ = 502 nm versus guest concentration.

NMR titration

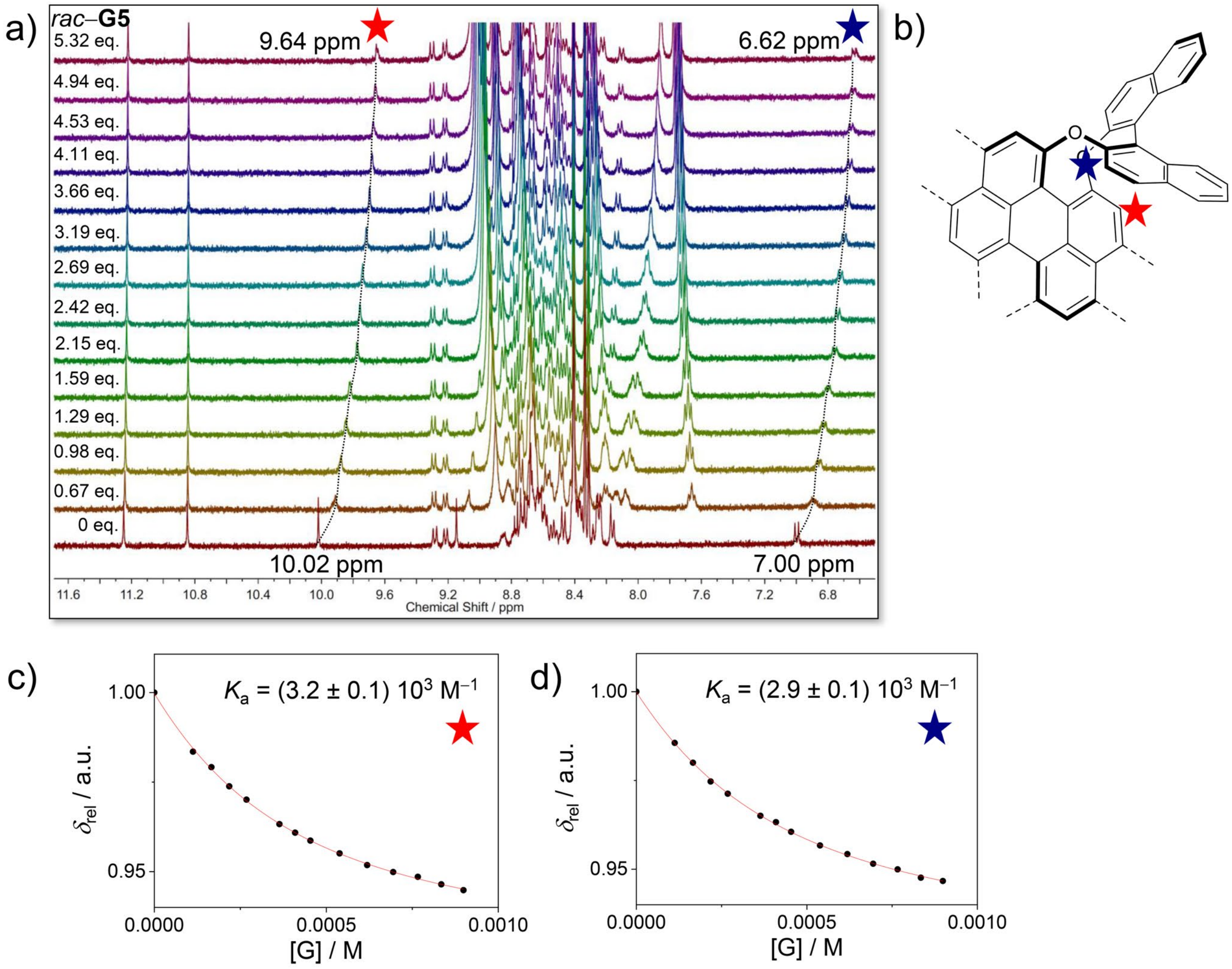

Figure S30. ^{1}H NMR spectra of *rac*-BPTI **7** in a MCH-d_{14} and toluene-d_8 mixture (24:1) at 293 K (c = 3.4 x 10^{-4} M) upon the addition of *rac*-[6]helicene (**G5**) as a guest, b) an excerpt of the molecular structure of BPTI **7** and the resulting plots of the normalized shift of c) a scaffold proton marked with a red star and d) a BINOL proton marked with a blue star with nonlinear curve fit (1:1 binding model, red curve).

Table S4. Summary of *bindfit*[S10] results for the UV/vis titration experiments of BPTI **7** upon addition of guests in MCH at 293 K.

**Host**	**Guest**	**K_a (UV/vis) [M^{-1}][a,b]**	**-ΔG_{293K} [kJ mol^{-1}][b,c]**
rac-BPTI **7**	naphthalene (**G1**)	80	10.7
rac-BPTI **7**	phenanthrene (**G2**)	1600	18.0
rac-BPTI **7**	*rac*-[4]helicene (**G3**)	5200	20.8
rac-BPTI **7**	*rac*-[5]helicene (**G4**)	6300	21.3
rac-BPTI **7**	*rac*-[6]helicene (**G5**)	10900	22.6
(*P*)-BPTI **7**[d]	(*P*)-[6]helicene (**G5**)	10700	22.6
(*P*)-BPTI **7**[d]	(*M*)-[6]helicene (**G5**)	550	15.4

[a] Global 1:1 fit from 428 nm to 550 nm as determined by *bindfit* global analysis. [b] Error of ±1 % as determined by *bindfit* global analysis. [c] Gibbs energy calculated by $\Delta G = -RT\ln(K_a)$, with T = 293 K. [d] Similar binding constants were obtained for the respective enantiomers with (*M*)-BPTI **7**.

Pictures of free (*P*)-BPTI 7, its homochiral complex with (*P*)-[6]helicene and its heterochiral complex with (*M*)-[6]helicene:

a)

b)

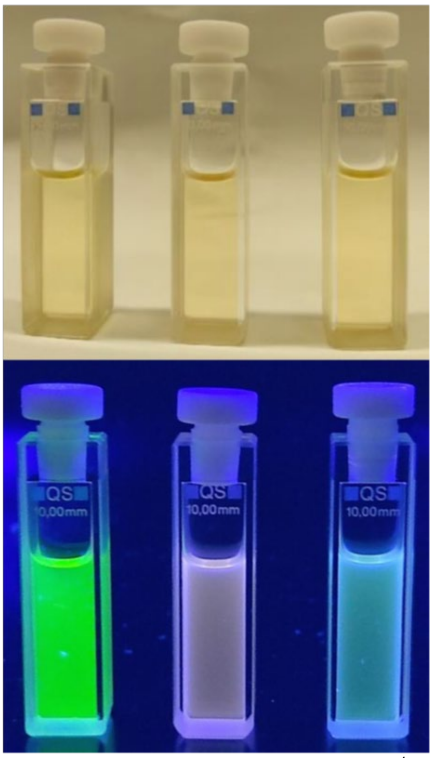

Figure S31. (*P*)-BPTI **7** (c = 8.6 x 10^{-6} M) without guest (left), with (*P*)-[6]helicene (middle, **G5**, c = 6.1 x 10^{-4} M) and with (*M*)-[6]helicene (right, **G5**, c = 5.8 x 10^{-4} M) in MCH at 293 K under a) sun light and b) UV light (375 nm).

Under sun light only small color differences can be observed between the solutions (slightly more orange for the homochiral complex). Under UV light, however, the color differences are clearly visible due to the exciplex-type fluorescence of the homochiral complex. The emission of (*P*)-BPTI **7** in MCH solution changes from yellow-green (Figure S31, left) to orange (Figure S31, middle) upon complex formation with (*P*)-[6]helicene. In contrast, the green emission color of the heterochiral mixture of (*P*)-BPTI **7** with (*M*)-[6]helicene (Figure S31, right) simply represents the sum of the yellow emission from BPTI **7** and the blue emission of the [6]helicene.

Supporting References

[S1] L. Li, Y.-J. Hong, D.-Y. Chen, M.-J. Lin, *Electrochimica Acta* **2017**, *254*, 255–261.
[S2] L. Li, H.-X. Gong, D.-Y. Chen, M.-J. Lin, *Chem. Eur. J.* **2018**, *24*, 13188–13196.
[S3] D. Meinhard, M. Wegner, G. Kipiani, A. Hearley, P. Reuter, S. Fischer, O. Marti, B. Rieger, *J. Am. Chem. Soc.* **2007**, *129*, 9182–9191.
[S4] Y. Nakai, T. Mori, Y. Inoue, *J. Phys. Chem. A* **2012**, *116*, 7372–7385.
[S5] P. Ravat, R. Hinkelmann, D. Steinebrunner, A. Prescimone, I. Bodoky, M. Juricek, *Org. Lett.* **2017**, *19*, 3707–3710.
[S6] G. Seybold, G. Wagenblast *Dyes Pigm.* **1989**, *11*, 303–317.
[S7] G. Sheldrick, *A short history of SHELX. Acta Crystallogr., Sect. A: Found. Crystallogr.* **2008**, *64*, 112−122.
[S8] G. Schoetz, O. Trapp, V. Schuring, *Electrophoresis* **2001**, *22*, 3185–3190.
[S9] L. Arrico, L. D. Bari, F. Zinna, *Chem. Eur. J.* **2021**, *27*, 2920–2934.
[S10] P. Thordarson, *Chem. Soc. Rev.* **2011**, *40*, 1305–1323; b) The program *bindfit* is available free of charge via http://supramolecular.org (01.21.2022).
